# The association between sedentary behaviour, physical activity and type 2 diabetes markers: A systematic review of mixed analytic approaches

**DOI:** 10.1371/journal.pone.0268289

**Published:** 2022-05-11

**Authors:** Francesca Romana Cavallo, Caroline Golden, Jonathan Pearson-Stuttard, Catherine Falconer, Christofer Toumazou

**Affiliations:** 1 Centre for Bio-Inspired Technology, Electrical and Electronic Engineering Department, Imperial College London, London, United Kingdom; 2 DnaNudge Ltd, London, United Kingdom; 3 Department of Epidemiology and Biostatistics, School of Public Health, Imperial College London, London, United Kingdom; 4 United Kingdom Health Security Agency, London, United Kingdom; PLOS, UNITED KINGDOM

## Abstract

The negative effect of sedentary behaviour on type 2 diabetes markers is established, but the interaction with measures of physical activity is still largely unknown. Previous studies have analysed associations with single-activity models, which ignore the interaction with other behaviours. By including results from various analytical approaches, this review critically summarises the effects of sedentary behaviour on diabetes markers and the benefits of substitutions and compositions of physical activity. Ovid Medline, Embase and Cochrane Library databases were systematically searched. Studies were selected if sedentary behaviour and physical activity were measured by accelerometer in the general population, and if associations were reported with glucose, insulin, HOMA-IR, insulin sensitivity, HbA1c, diabetes incidence, CRP and IL-6. Forty-five studies were included in the review. Conclusive detrimental associations with sedentary behaviour were determined for 2-h insulin (6/12 studies found associations), fasting insulin (15/19 studies), insulin sensitivity (4/6 studies), diabetes (3/4 studies) and IL-6 (2/3 studies). Reallocating sedentary behaviour to light or moderate-to-vigorous activity has a beneficial effect for 2-h glucose (1/1 studies), fasting insulin (3/3 studies), HOMA-IR (1/1 studies) and insulin sensitivity (1/1 studies). Compositional measures of sedentary behaviour were found to affect 2-h glucose (1/1 studies), fasting insulin (2/3 studies), 2-h insulin (1/1 studies), HOMA-IR (2/2 studies) and CRP (1/1 studies). Different analytical methods produced conflicting results for fasting glucose, 2-h glucose, 2-h insulin, insulin sensitivity, HOMA-IR, diabetes, hbA1c, CRP and IL-6. Studies analysing data by quartiles report independent associations between sedentary behaviour and fasting insulin, HOMA-IR and diabetes only for high duration of sedentary time (7–9 hours/day). However, this review could not provide sufficient evidence for a time-specific cut-off of sedentary behaviour for diabetes biomarkers. While substituting sedentary behaviour with moderate-to-vigorous activity brings greater improvements for health, light activity also benefits metabolic health. Future research should elucidate the effects of substituting and combining different activity durations and modalities.

## Introduction

Sedentary behaviour (SB), defined as any activity below 1.5 Metabolic Equivalent (MET) in either a lying, reclining or sitting position [1], is unfavourably associated with several T2D biomarkers independent of moderate-to-vigorous activity (MVPA) [2, 3]. Despite many countries having introduced guidelines on SB, such as UK [4] and Australia [5], a recent paper [6] summarising the research behind the newest WHO guidelines [7] highlighted the lack of sufficient evidence to set time-based recommendations regarding SB. Additionally, the paper emphasised the need to establish alternative ways to offset the damaging effects of SB, given that the high levels of MVPA (> 300 minutes/week) needed to reduce the mortality risk—as suggested by the current evidence—may be unfeasible for a large part of the population. However, while there is proof of the benefits of MVPA on health outcomes, less clear is the effect of low-intensity physical activity (LIPA). Several works document the benefits of LIPA for markers of T2D (e.g. [8–10]), but many studies analysing the cross-sectional association between SB and diabetes markers have not included LIPA in the analysis, given the high collinearity between the two variables, which prevents them from being included simultaneously in regression models. A review by Chastin et al. [11] found that LIPA improves cardiometabolic health, but did not include information on the relationship with SB. Amagasa et al. [12] reviewed the effects of LIPA on cardiometabolic biomarkers and found positive associations independently of MVPA, but could not find evidence of the combined effects of time spent in PA and SB. Studies using the isotemporal substitution model (ISM) [13] or a compositional transformation [14] have been able to include LIPA in the models and reported benefits associated with the reallocation of SB to LIPA. Previous reviews [15–18] summarised the evidence around the reallocation of time between SB and PA in the adult population, but did not address whether there is a minimum and maximum allocation time of LIPA and MVPA for which such benefits are observed.

This review has three aims. Firstly, to summarise the current evidence on the effects of SB on T2D biomarkers, accounting for MVPA, in the general healthy adult population. Previous systematic reviews [2, 19] have analysed the association between device-measured SB and cardiometabolic biomarkers, but did not exclude studies that did not account for MVPA in the analysis. Given that the relationship between SB and health outcomes changes depending on the MVPA level [20], accounting for MVPA is crucial to obtain reliable results. Secondly, this review aims to assess how different time allocations and compositions impact the magnitude of the reduction in T2D markers. Thirdly, we compare the findings obtained with different analytical approaches, namely linear regression, linear regression by quartiles, ISM and compositional transformation. To our knowledge, this is the first review to compare the association between SB and biomarkers of T2D obtained with different analytical methods.

## Methods

**Fig 1:**
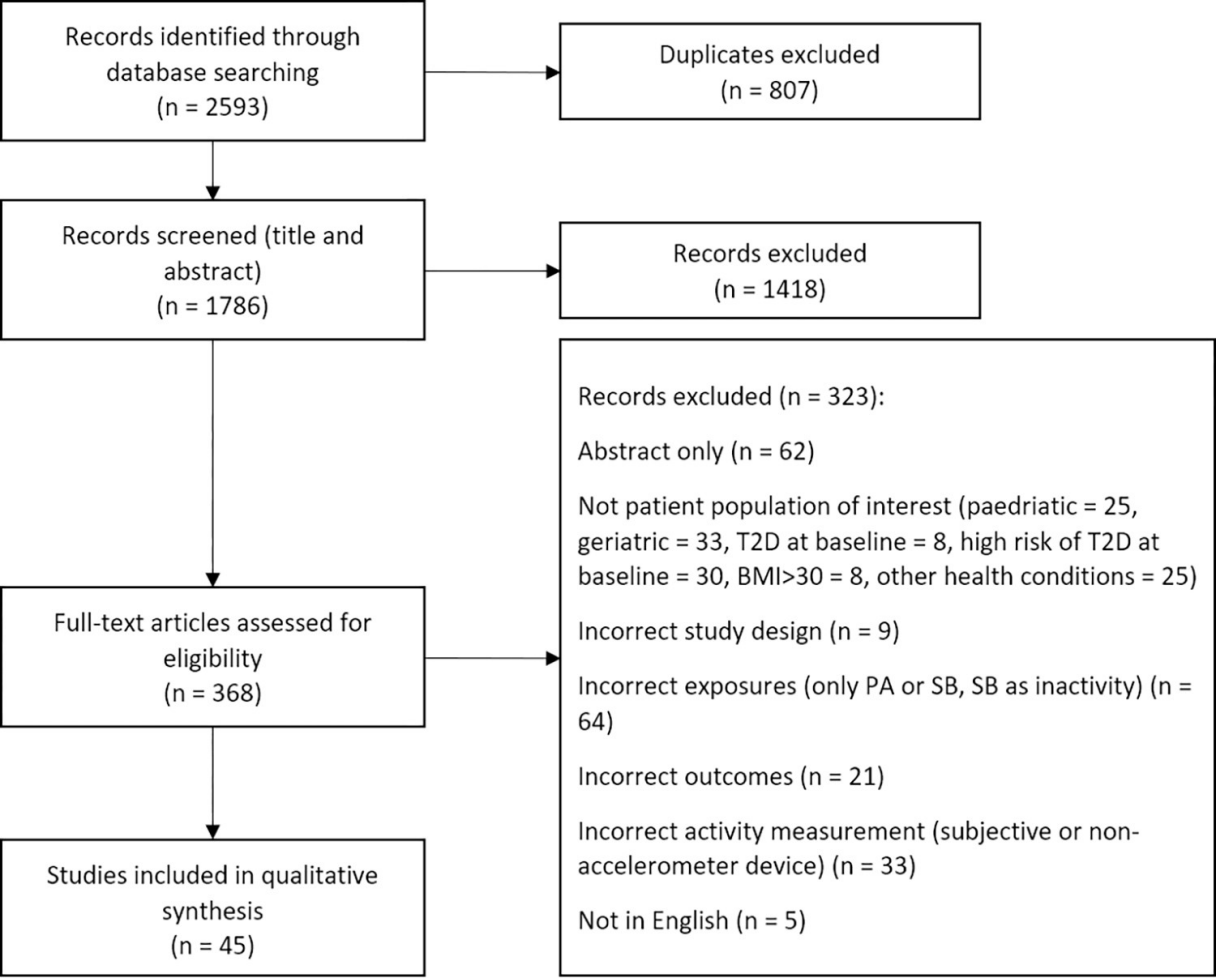
PRISMA flow diagram of study selection process.

The PRISMA guidelines [21] were followed to conduct and report the results of this review (see Fig 1).

### Search strategy and inclusion criteria

We conducted the search strategy following PEO (population, exposure, outcome) framework [22]. The population of interest was the general adult population (aged 18–65), without pre-existing conditions; the exposure was defined as accelerometer-measured physical activity or sedentary behaviour; and the outcomes were the following: fasting insulin, fasting glucose, insulin sensitivity, HbA1c, HOMA-IR, 2h glucose, 2h insulin, incident diabetes, CRP and IL-6.

We searched Ovid Medline, Embase and Cochrane Library for relevant publications, with no specified beginning or end date of publication, on the 15th June 2021. The search term used can be found in the S1 File.

For inclusion, each study had to meet the following criteria:

1. In English
2. Longitudinal or cross-sectional design
3. Adult general population (18 ≥ years of age ≤ 65)
4. Device-measured physical activity and sedentary time by accelerometer
5. At least one diabetes marker of interest (fasting insulin or glucose, insulin sensitivity, HbA1c, HOMA-IR, 2h glucose, 2h insulin, CRP, IL-6)
6. Both physical activity and sedentary time reported
7. SB adjusted for MVPA, if standard regression was used
8. Association between physical activity, sedentary time and T2D markers reported

Exclusion criteria included: study on non-general population (diabetic, pre-diabetic, BMI > 30, with metabolic syndrome, pregnant, with pre-existing conditions), sedentary behaviour defined as not meeting the activity guidelines (≥ 150 mins/week), physical activity and sedentary time assessed by subjective methods or devices other than accelerometer (such as pedometer).

FRC and CG screened all titles and abstracts for the studies obtained through the electronic search, and the studies meeting the inclusion criteria underwent full text review. Any discrepancies were discussed between FRC and CG both at the title/abstract screening stage and the full text review stage. Disagreements were resolved with consensus at both screening stages. The Covidence systematic review software was used for the study selection.

### Quality assessment

Each of the selected papers was assessed for quality on a scale from 0 to 7, where scores ≤ 3 represent poor quality, 4–5 medium quality and ≥ 6 good quality. The quality assessment tool was developed based on the Newcastle-Ottawa Scale [23] and adapted following the scale developed by Brocklebank et al. [2]. The Newcastle-Ottawa Scale was developed for prospective studies and thus, many criteria were not applicable to the studies included in the review (for e.g., “Demonstration that outcome of interest was not present at start of study” and “Adequacy of follow up of cohorts”). Therefore, additional criteria from Brocklebank et al. [2] were integrated to reflect the key characteristics of the selected studies. Two points were available for selection of the cohort (representativeness and description), one for measurement of exposure (at least 4 valid days of accelerometer data), three for comparability (adjusted for BMI and/or waist circumference; for sex, age and ethnicity; and for accelerometer wear time) and one for data analysis (if points estimates and measures of variability were reported). The quality assessment was done by FRC and verified by CG.

### Data extraction

We extracted author, population, study type, observation period (if applicable), sample size, mean age, outcomes, covariates (gender, age, BMI, diet, etc.), accelerometer device type, unit of exposures (PA and SB), statistical method, and effect measure for each study. Cut-offs and units (hours/day, total time, etc.) were recorded for the exposures. The primary outcome was the association between sedentary behaviour and T2D markers, adjusted or substituted with different PA intensities. Due to the heterogeneity between the studies–especially regarding devices and activity cut-offs–no meta-analysis could be conducted, and the data is presented qualitatively.

The significance of the associations was determined based on the criteria by Sallis et al. [24] for which there is an association between exposures and outcome only if more than 60% of the studies report a significant association (the association is indeterminate if 34–59% report it, and no association if 0–33% report it).

The data extraction was done in Microsoft Excel by FRC and verified by CG.

## Results

The initial search identified 2593 studies, 46 of which were included in the review. Three studies had a prospective design [25–27]. There was high heterogeneity between the studies (see Table 1). A wide array of monitoring devices was used, with Actigraph being the most common; 11 studies applied activities cut-offs as Metabolic Equivalent (MET), while 34 studies as counts-per-minute (cpm); among the latter, SB was generally classified as < 100cpm, while for MVPA a wide array of values was used (760cpm, 1040cpm, 1486cpm, 1535cpm, 1952cpm, 2020cpm, 2690cpm). Most studies reported sedentary time as hours or minutes per day, while activity was mostly reported as a percentage of the total recorded time. Fasting glucose, fasting insulin and HOMA-IR were the most frequently reported outcomes. 14 studies used a population that was free of existing metabolic conditions (four of which needed to do so as they reported on diabetes incidence [25, 28–30]) and 20 studies adjusted for BMI and/or waist circumference, gender, age, ethnicity and MVPA simultaneously (see S3 Table in S1 File). Eight studies used the ISM, four used a compositional transformation, 14 reported mean differences, and the rest evaluated associations with either linear or logistic regression, reporting Odds Ratios (OR), Risk Ratios (RR) or regression coefficients. Of the selected studies, seven had a high-quality rating (score of 7 or 6) and 14 had a low-quality rating (score ≤ 3). A complete description of the studies included can be found in Table 2, while a detailed quality assessment can be found in S3 Table in S1 File.

**Table 1:** Characteristics of the selected studies.

Study design	n
Cross sectional	42
Prospective	3
**Device type**	n
Actigraph	24
Actiheart	2
Actical	6
ActiTrainer	1
Vitamove	1
ActivPal	4
Active style	3
GENEActiv	1
Hookie	1
Sensewear	2
**Sedentary time quantification**	n
Hours or minutes/day	23
Percentage time	4
30 minutes/day bouts	5
Total time	3
Other	10
**PA time quantification**	n
Hours or minutes/day	24
Percentage time	2
30 minutes bouts/day	4
Minutes/week	1
Total time	3
Other	11
**Outcomes**	n
Fasting Glucose	32
fasting insulin	19
Insulin sensitivity	6
Diabetes incidence	4
HOMA-IR	14
HbA1c	6
2h glucose	9
2h insulin	3
CRP	14
IL-6	3
**Statistical analysis**	N
Isotemporal substitution	8
Compositional transformation	4

**Table 2:** Summary of the selected papers.

Study	Study type	Sample size	Mean age (SD)	PA exposure	SB exposure	SB-PA cut-offs	Device	Device placement	Outcomes	Variables adjusted for	Statistical method	Effect measure	Quality
**Bakrania 2016 [31]**	Cross sectional	2131	50.8 (0.47)	> 150 min/week	Mins/day	100–1952 cpm	Actigraph GT1M	Right hip	BMI, WC, HDL-C, total cholesterol, HbA1c	Adjusting for age; BMI (except in the model with BMI as the dependent variable); cardiovascular disease index; ethnicity; fruit and vegetable consumption; sex; smoking status; socio-economic status; and accelerometer wear-time. Models for HDL-cholesterol and total cholesterol were also controlled for BP medication and cholesterol medication. The model for HbA1c was controlled for prescribed medication.	Linear regression	Regression coefficients.	7
**Balkau 2008 [32]**	Cross sectional	801	43 ± 9 men, 45 ± 8 women	Average counts/min	% time	100–1952 cpm	Actigraph, AM7164-2.2;	Small back	Insulin-sensitivity	BMI, WC, fasting glucose, alcohol intake, smoking, diabetes in family, and menopause.	Mixed linear models	Regression coefficients	4
**BaroneGibbs 2015 [25]**	Longitudinal	1,474 for HbA1c n = 1,317 for 2-h glucose.	45	Mins/day	Hours/day	100–2020 cpm	Actigraph 7164	Waist	Fasting glucose, 2h-glucose, fasting insulin, HOMA-IR, HbA1c. Diabetes incidence, prediabetes by Hb1Ac incidence, impaired fasting glucose and impaired glucose tolerance incidence.	Age, ethnicity, centre, sex, education, income, diabetes, BMI, hypertension, cholesterol, smoking, alcohol, and accelerometer wear time. For longitudinal analysis, baseline values were added as a covariate.	Linear and logistic regression	Regression coefficient % difference associated with each additional 1 h increase in SB	5
**Buman 2014 [33]**	Cross sectional	2185 for the full sample, 923 for fasting subsample	46.6 (18.5)	30 Mins/day	30 Mins/day quartiles	100–1952 cpm	Actigraph 7164	Right hip	WC, HDL-C, TG (fasting) and fasting insulin, LDL, glucose, HOMA-S (sensitivity), HOMA-β	Adjusted for age, sex, ethnicity, marital status, education, work status, income; smoking, depressive symptoms, total energy intake, saturated fat, caffeine, alcohol; general health rating, diagnosis of cancer, CVD, diabetes; current use of diabetic, antihypertensive, lipidemic or CVD medication, total assessment time (wear time).	Linear regression. Isotemporal substitution models.	Relative risk	4
**Carson 2014** [34]	Cross sectional	2551	46	Hours/day	Hours/day	100–1535 cpm	Actical	Right hip	WC, BP, HDL-C, CRP, TG, LDL-C, glucose, fasting insulin	Adjusted for MVPA, age, sex, income, smoking, alcohol use, BP medication, T2D history, heart disease, cancer, survey cycle.	Linear regression.	Unstandardized regression coefficients	3
**Celis-Morales 2012 [35]**	Cross sectional	317	37.5 ±12.8	Mins/day	Mins/day	100–1952 cpm	ActiTrainer	Left hip	Fasting insulin, glucose, TG and total, LDL-C and HDL-C and HOMA-IR	Adjusted for age, sex, ethnicity, environment (rural or urban), socio-economic status and smoking. And for other activity levels.	Linear regression.	Means ± SE and regression coefficients	4
**Chastin 2015 [14]**	Cross sectional	1937	43	Portion of the day	Portion of the day	100–1952 cpm	Actigraph 7164	Hip	BMI, WC, BP, HDL-C, total cholesterol, glucose, insulin, TG, HOMA-IR, CRP	Age, sex, ethnicity; marital status; education, work status, family income to poverty level, smoking status, consumption of caffeine and alcohol, total energy and saturated vat dietary intake. Self-reported health, medication use. Fasting variables adjusted for BMI.	Linear regression with and without compositional transformation (isometric log-ratio).	Regression coefficients	5
**Debache 2019 [36]**	Cross sectional	131	50.6 ± 9.6	Not reported	Not reported	Not reported	Vitamove Research-V1000	Trunk and right upper leg	Glucose, LDL-C, HDL-C, TG, log(BMI), WC	Sex, age, annual income, education, and two nutritional indexes (first two dimensions of principal components analysis with variables relating to health and dietary habits), BMI	Linear regression with and without compositional transformation (isometric log-ratio). Isotemporal substitution models.	Regression coefficients	4
**Diaz 2017 [37]**	Cross sectional	12083	Q1: 39.6 (38.8–40.4), Q2: 41.1 (40.3–41.9), Q3: 40.8 (39.9–41.6), Q4: 43.2 (42.1–44.3)	Mins/day	Mins/day	100–1535 cpm	Actical version B-1	Right hip	2h-glucose, HbA1c, insulin, HOMA-IR	Age, sex, study centre, Hispanic background (ethnicity), education level, annual household income, employment status, birthplace outside the US, smoking, alcohol drinking, alternative healthy eating index–2010 score, short-Form 12 health survey physical score, short-Form 12 Health Survey mental score, hypertension, estimated glomerular filtration rate <60 mL·min−1·1.73 m−2, high-sensitivity CRP, antidiabetic medication, health insurance, healthcare use, MVPA, mean sedentary bout duration.	Linear regression by quartiles of SB.	Marginal means per quartile	3
**Edwards 2018 [40]**	Cross sectional	627	33.8 (0.3)	Above/below sample median of 30.3 Mins/day	Above/below median of 437.5 Mins/day	100–2020 cpm	Actigraph 7164	Not reported	Elevated CRP	Age, sex, ethnicity, serum cotinine, quality of life, diabetes, BP or cholesterol medication, multimorbidity	Logistic regression.	Odds ratios	3
**Ekblom-Bak 2015 [38]**	Cross sectional	836	57.5 (54–61.8)	Mins/day	Mins/day	200–2690 cpm	Actigraph GT3X and GT3X+	Right hip	MS (metabolic syndrome), TG, glucose, BP, WC, HDL. All expressed as incidence (high/low)	Sex, age, education (2 groups), smoking (2 groups), psychological stress (4 groups), daily energy intake (quartiles) VO2max. Wear time and other PA intensity levels.	Isotemporal substitution models.	Odd ratios	5
**Ekblom-Bak 2016 [39]**	Cross sectional	654	57 (54–61)	Mins/day	Mins/day	200–2690 cpm	Actigraph GT3X and GT3X+	Right hip	Fasting glucose, fasting insulin, HOMA-IR, WC, VO2 max, fasting glucose (high vs low)	Adjusted for sex, age, education, smoking, psychosocial stress, total wear time.	Isotemporal substitution models.	Relative rate (% shift in the mean value)	4
**Elhakeem 2018 [41]**	Cross sectional	1622	60 to 64	Hours/day	Hours/day	1.5–3 MET	Actiheart	Chest	CRP, IL-6, e-selectin, Tissue plasminogen activator, leptin, adiponectin	Socio-economic position, smoking, long-term illness, health problems or disability, BP, diabetes, CVD, medication use, education, age, adiposity. PA parameters were adjusted for wear time and diurnal information bias.	Linear regression.	Mean % difference	2
**Farrahi 2021 [42]**	Cross sectional	3443	46.6 (0.5)	Mins/day	Mins/day	1.5-3-6 MET	ActiGraph GTX3	Hip	2h insulin, fasting glucose, total/HDL-C ratio, LDL-C/HDL-C ratio, body fat, fat mass, waist circumference, fasting insulin,	Age, sex, birth weight, education level, employment status, marital status, household income, health-related quality of life, lifestyle factors (smoking status and alcohol consumption), and medication (for BP, cholesterol and diabetes).	Linear regression with compositional transformation	Regression coefficients	3
**Farrahi 2021 [43]**	Cross sectional	5840	46.6 (0.6)	Quartiles based on minutes accumulated during sedentary breaks	Quartiles based on frequency of bouts (1–5 min, 5–10 min, 10–15 min, 15–30 min).	1.5–3 MET	Hookie AM20; Traxmeet Ltd.	Hip	2h insulin, fasting insulin, triglycerides, total/HDL-C, LDL/HDL-C, 2h glucose, fasting glucose.	Model 1 adjusted for age, sex, education, employment, and marital status; Model 2 further adjusted for medication use, health-related quality of life score, smoking, alcohol consumption, and income. Model 3 additionally adjusted for total sedentary time, and Model 4 for total MVPA time.	Linear regression	Regression coefficients	3
**Garcia-Hermoso 2015 [44]**	Cross sectional	1122	55 ± 13.6	Mins/day quartiles	Mins/day quartiles	100–2020 cpm	Actigraph GT3X	Right waist	BMI, WC, TG, HDL-C, HOMA-IR, mean arterial pressure	Adjusted for age, smoking habit, drinking habit, and accelerometer wear time; TG, HDL-C and TG/HDL-C ratio was additionally adjusted for the use of antihypertensive or lipid-lowering drugs (yes/no) and MVPA. Stratified by sex.	ANCOVA	Mean differences)	4
**Gennuso 2014 [45]**	Cross sectional	5076	43.8 ± 19.5	Mins/week	Hours/day	1000–760 cpm	Actigraph 7164	Right hip	MS, high WC, high TG, low HDL, high fasting glucose, high BP	For regression analyses: age, sex, ethnicity, education, marital status, family income, smoking status, monitor wear time. For secondary hypothesis: age, sex, PA status (age and PA divided in 3 and 4 categories respectively)	Logistic regression with and without natural cubic splines.	Odd ratios	4
**Gradmark 2011 [46]**	Cross sectional	73	28.6 (4.4)	% wear time	% of wear time	0 cpm	Actiheart	Left side of chest	Matsuda composite insulin sensitivity index, oral disposition index, first phase insulin response	Age, PAEE, age, weight, accelerometer wear-time.	Linear regression.	Spearman correlation coefficient and R^2^ with linear regression	5
**Healy 2007 [47]**	Cross sectional	173	53.3 (51.5–55.1)	Hours/day	Hours/day	100–1952 cpm	Uniaxial Actigraph WAM 7164	Right anterior axillary line	2h-glucose, fasting glucose.	Age, sex, accelerometer wear time, height, WC, alcohol intake, education, income, smoking status, family history of diabetes. Sedentary time was also adjusted for MVPA in model 3	Linear regression.	Non-standardized regression coefficient	5
**Healy 2011 [48]**	Cross sectional	4757 (2118 fasting analysis, 910 for 2h-glucose)	46.5 (14.2)	Hours/day	Hours/day	100–1952 cpm	Actigraph 7164	Right hip	WC, blood pressure, HDL-cholesterol, CRP, triglycerides, fasting glucose, fasting insulin, HOMA-%B, HOMA-%S (sensitivity), 2h-glucose.	Age, sex, and race/ethnicity, socio-demographic, behavioural and medical factors, quartiles of MVPA, WC, sedentary time, wear time.	Linear regression.	Mean per quartile of SB with p-value for trend	6
**Healy 2015 [49]**	Cross sectional	741	57 (median)	Addition of 2Hours/day	Addition of 2Hours/day	1.4–3 MET	ActivPal 3	Right anterior thigh	BMI, WC, BP, fasting plasma glucose, HbA1c, HDL-C, LDL-C, TG, 2h-glucose	Age, sex, BP/cholesterol/diabetes medication, ethnicity, present occupation or previous if not working, household income, employment status, fibre intake, energy intake, energy-adjusted fibre intake, alcohol intake, sodium intake, potassium intake, fruit and vegetable serves, wear time.	Isotemporal substitution models.	Regression coefficients	4
**Honda 2014 [50]**	Cross sectional	661	43± 9 yrs	MET Hours/day	Hours/day tertiles	1.5 MET or 100cpm for SB	Active Style pro HJA 350-IT	Not reported	BMI, WC, BP, TG, HDL-C, blood glucose, HbA1c	Sex, age, education, smoking and drinking habits, marital status, occupation. Calorie intake, saturated fat consumption, use of medications, depressive symptoms, MVPA, wear time.	Linear regression.	Regression coefficients corresponding to the mean difference per 60 minutes/day greater sedentary time	5
**Honda 2019 [29]**	Cross sectional	1758	split by sedentary groups. From lowest to highest: 61(0.5), 61(0.4), 59.7(0.5), 63.6(0.8)	Not reported	Hours/day. Split into 4 groups (<6, 6–8, 8–10, >10)	1.5–3 MET	Active Style pro HJA 350-IT	Not reported	Diabetes incidence, HOMA-IR	Age, sex, accelerometer wear time, family history of diabetes, hypertension, total cholesterol, HDL-C, TG, smoking, alcohol intake, MVPA.	Linear and logistic regression.	Odds ratios for diabetes, geometric means for HOMA-IR.	5
**Kim 2013 [51]**	Cross sectional	483	47.9±9	MET-hours/day (tertiles)	Hours/day	1.5–3 MET	HJA-350IT active style pro	Right hip	WC, systolic BP, change in BP, fasting glucose, TG, HDL-C, metabolic syndrome	Energy intake, smoking status, antihypertensive and antidyslipidemic drugs. Regression adjusted for age, sex, smoking, calorie intake, wear time, MVPA.	Generalised linear models and logistic regression.	Means, regression coefficients.	5
**Knaeps 2016 [52]**	Cross sectional	341	53.8 ± 8.9yrs	Hours/day	Hours/day	1.5–3 MET	Sensewear armband	Not reported	Cardiometabolic risk score, WC, fasting glucose, HDL, TG, BP	All models adjusted for age, sex, waking time, smoking, alcohol, sugar and fat, education level. Model 2 further adjusted for SB and MVPA. Model 3 is fully adjusted.	Linear regression and Pearson correlations.	Correlation and standardized regression coefficients	2
**Lahjibi 2013 [26]**	Cross sectional and prospective	727	43±9 men, 45±8 women	Total hours	Total hours	100–1952 cpm	Actigraph AM7164	Small back	BMI, fat, WC, BP, heart rate, total cholesterol, HDL-C, LDL-C, TG, fasting glucose, 2h-glucose, fasting insulin, 2h insulin, insulin sensitivity, HOMA-IR, insulin secretion index	Age, sex, recruiting centre, SB was adjusted for MVPA, wear time.	Linear models.	Means for SB quartile, 3-year change in fasting insulin and glucose and HOMA-IR	4
**Loprinzi 2014 [53]**	Cross sectional	5580	not specified	> or < 150 min/week	LIPA-SB balance (>/<1)	100–2020 cpm	Actigraph AM7164	Not reported	BMI, WC, CRP, white blood cells, neutrophils, HDL-C, total cholesterol, LDL-C, TG, glucose, fasting insulin, homocysteine	Age, sex, ethnicity, cotinine, poverty-to-income ratio, BMI, comorbidity index, wear time, drug therapy.	Linear regression.	Regression coefficients	5
**Lynch 2011 [54]**	Cross sectional	467 in the fasting sample. 1024 in the non-fasting sample	62.4 (9.5)	Minutes/day	Hours/day	100–1952 cpm	Actigraph AM7164 (1s)	Not reported	BMI, WC, CRP (non-fasting). HOMA-IR, fasting plasma glucose, fasting insulin	Age, ethnicity, education, marital status, energy and alcohol intake, sedentary and LIPA were adjusted for MVPA, MVPA adjusted for SB, reproductive health data. One sex. Model 3 (fasting) additionally adjusted for WC. Data corrected for wear time.	Linear regression.	Marginal means per quartile	7
**Maher 2014 [55]**	Cross sectional	4618	28.6 (women), 33.2 (men)	Total minutes	Total hours	100–2020 cpm	Actigraph AM7164 (1s)	Right hip	WC, log(BP), log(HDL-C), log(CRP), log fasting TG, log(fasting plasma glucose), log (fasting insulin), log (HOMA-S), log(glucose tolerance test), 2h-glucose	Age, sex, ethnicity, household income, education, family history of stroke/hypertension, of cancer, of CVD, of diabetes. Use of medications, smoking, energy intake, saturated fat intake, accelerometer wear time.	Linear regression.	Regression coefficients	4
**McGregor 2018 [56]**	Cross sectional	6322	41.3 (0.2) full sample, 41.8 (0.8) fasting sample	Not reported	Not reported	100–1535 cpm	Actical (60s)	Not reported	BMI, waist circumference, aerobic fitness, blood pressure, resting heart rate, HDL-C, LDL-C, triglycerides, fasting insulin, glucose, CRP, grip strength, self-assessed mental health	Age, sex, education, smoking status, alcohol consumption, chronic condition, self-rated health.	Linear regression with compositional transformation.	Compositional regression coefficients	3
**Mossavar-Rahmani 2020 [57]**	Prospective	8049	Not reported	Meeting the 150 min/week of MPA or 75 min/week of VPA guidelines.	Quartiles of SB with cut-offs 10.8, 12, 13, 16 h/day.	100-1535-3961 cpm	Actical	Right iliac crest	BMI, waist circumference, blood pressure, LDL-C, HDL-C, triglycerides, fasting glucose, 2h glucose, fasting insulin, HOMA-IR.	Model 1 adjusted for age, sex, use of medications, baseline levels of the dependent variable, and elapsed time between visits. Model 2 further adjusted for baseline household income, education, employment status, Hispanic/Latino background, field center, and nativity status, smoking, alcohol consumption, health insurance status, healthcare utilisation, self-reported health, diet quality and change in health insurance. Model 3 adjusted for Model 2 covariates and sedentary time in models of MVPA or MVPA in models of sedentary time. Data corrected for wear time using weighting technique.	Linear regression.	Adjusted means	3
**Parsons 2017 [58]**	Cross sectional	1274	78.4 ± 4.6	Total 10 mins/day	Total 30 mins/day	100–1040 cpm	Actigraph GT3X	Right hip	IL-6, CRP, tissue plasminogen activator, Von Willebrand factor, D-dimer, insulin-like growth factor 1	PA and SB were mutually adjusted. One sex and one ethnicity. All models adjusted for BMI, wear time, season, hour of blood sampling, age, region of residence, social class, living alone, smoking status, alcohol consumption.	Linear regression.	Regression coefficients. Results are the % difference in biomarker levels.	5
**Peterson 2014 [59]**	Cross sectional	5268	High MVPA: 39.75 (0.51) (low SB), 42.56 (0.75) (moderate SB), 42.85 (0.65) (high SB). Moderate MVPA: 44.40 (0.75) (low SB), 46.07 (0.57) (moderate SB), 48.77 (0.61) (high SB). Low MVPA: 56.27 (1.33) (low SB), 62.23 (0.93) (moderate SB), 63.60 (0.74) (high SB).	Average minutes/day	Average minutes/day	100–2020 cpm	Actigraph 7164	Right hip	Fasting glucose, fasting insulin, HOMA-IR, TG, CRP, total cholesterol, HDL-C, BP	None	Linear and logistic regression.	Unadjusted means per MVPA and SB groups	3
**Peterson 2015 [28]**	Cross sectional	2816	Divided for sex and BMI	Tertiles, cut-offs not specified	Tertiles, cut-offs not specified	100–2020 cpm	Actigraph AM7164 (1s)	Right hip	Insulin resistance and diabetes	Ethnicity, level of education, % body fat, LIPA, MVPA. Stratified by sex.	Logistic regression.	Odd ratios for IR and diabetes.	5
**Phillips 2017 [60]**	Cross sectional	396	59.58 ± 5.46	30 mins/day	30 mins/day	1.5–3 METs	GENEActiv (60s)	Wrist	Complement C3, white blood cells, IL-6, leptin, adiponectin	Age, sex, smoking status, alcohol and energy intake, BMI, anti-inflammatory medication use. MVPA and total wear time included in models.	Linear regression and isotemporal substitution models.	Regression coefficients.	5
**Qi 2015 [61]**	Cross sectional	12083	Split by SB quartiles: 39 (38–40), 40 (39–41), 41 (40–42), 45 (44–46)	Mins/day	Hours/day (quartiles)	100–1535 cpm	Actical B1 (60s)	Above iliac crest	BP, LDL, HDL, TG, fasting glucose, 2h-glucose, fasting insulin, HOMA-IR, CRP	Age, sex, household income, education, employment status, ethnicity background, field centre, smoking, alcohol consumption, health insurance, healthcare use, self-reported health, diet quality, medications specific to each marker, MVPA, BMI, waist-hip ratio, noncompliance with device wear protocols.	Linear and logistic regression.	Adjusted means, adjusted means split by meeting/non-meeting the PA guidelines.	4
**Scheers 2013 [62]**	Cross sectional	370	41.7 ± 9.8	Hours/day	Hours/day	1.5–3 METs	SenseWear Pro 3 Armband	Over right tricep	Metabolic syndrome, obesity, hypertriglyceridemia, hypertension, hyperglycaemia (fasting glucose >100mg/dL)	Sex, age, education, smoking status, and alcohol consumption, and MVPA	Logistic regression.	Odd ratios	3
**Spartano 2017 [63]**	Cross sectional	2109	46.3 ± 8.9	20-minute bouts/day	% time, 5% increase	200–1486 cpm	Actical 198-0200-00 (60s)	Hip	HOMA-IR, IGF-1, high sensitivity CRP, adiponectin, leptin, SOB-R, leptin/SOB-R, FABP4, RBAP4	Cohort, age, sex, BMI, CVD, hypertension, current smoking, season of examination, residence, overnight wear, MVPA. One predominant ethnicity (European)	Linear regression.	Regression coefficient	7
**Stamatakis 2012 [64]**	Cross sectional	971	43.9 (13.5)	Mins/day	Mins/day	100–2020 cpm	Actigraph model GT1M,	Not reported	HDL-C, HbA1c, total cholesterol, BMI, WC, BP	Age, sex, social class, employment status, alcohol consumption, fruit and vegetable consumption, unhealthy eating index, psychological distress, cardiovascular or diabetes medication, occupational physical activity, MVPA, wear time.	Linear regression.	Unstandardised regression coefficients	3
**Stubbs 2017 [65]**	Cross sectional	199	44(9.9)	Steps/day	Minutes/day	100 cpm	Actigraph	Non dominant wrist	WC, BP, TG, HDL-C, fasting glucose	Age, sex, smoking habits, alcohol consumption, education, medications, positive and negative syndrome scale, steps	Linear regression.	Regression coefficient	1
**Van der Velde 2015 [30]**	Cross sectional	543	32.19 (0.57)	% time of spent in a day	% of time spent in a day	100–2020 cpm	ActiGraph AM-7164	Right hip	WC, BMI, BP, fasting glucose, HDL-C, triglycerides, CRP	Age, sex, ethnicity, health status, smoking status, BMI, MVPA	Lineal regression.	Standardized regression coefficient	6
**Van der Velde 2018 [66]**	Cross sectional	1933	59.7 (8.1)	1 SD	1 SD	Not reported	ActivPal 3	Right thigh	Diabetes and prediabetes incidence and metabolic syndrome	Age, sex, waking time, education, smoking, alcohol consumption, CVD history, mobility limitations, energy intake, body fat.	Logistic regression.	Odd ratios	3
**Varela-Mato 2017 [67]**	Cross sectional	159	50.0 (24.0, 67.0)	30 Mins/day	30 Mins/day	1.5–3 MET	ActivPal 3	Right thigh	WC, BMI, BP, fasting glucose, TG, HDL-C, LDL-C, total cholesterol	Age, ethnicity, education, shift pattern, smoking, alcohol intake, fruit and vegetable consumption, BMI. Wear time was included in the models.	Isotemporal substitution models.	Regression coefficients.	5
**Whitaker 2019 [27]**	Prospective	1922	45.3 ± 3.5	30 Mins/day	30 Mins/day	100–1952 cpm	Actigraph 7164 (60s) and Actigraph wGT3X-BT	Belt	WC, BP, glucose, insulin, TG, HDL-C, composite risk score	Age, sex, ethnicity, years of education, employment status, health insurance, meds use for BP cholesterol and diabetes, smoking status, alcohol consumption, BMI, field centre. Wear time was included in the models.	Isotemporal substitution models.	Regression coefficients,	6
**Zheng 2020** [68]	Cross sectional	94	21.7 (3.38)	hours/day	hours/day	MVPA > 5123 cpm	ActivPAL	Right thigh	Triglycerides, total cholesterol, LDL-C, HDL-C, glucose, insulin, C-peptide, HOMA-IR, leptin, resistin, adiponectin, E-selectin, P-selectin, VCAM-1, ICAM-1.	Model 1 was adjusted for age, BMI, activPAL wear time, family history of diabetes, family history of CVD, and ST (sedentary breaks only). Model 2 was additionally adjusted for MVPA.	Generalised linear models	Regression coefficients	7

BMI: body mass index; BP: blood pressure; CPM: counts-per-minute; CRP: c-reactive protein; FABP4: fatty acid-binding protein 4; HbA1c: haemoglobin A1c; HDL-C: High-density lipoprotein cholesterol; HOMA-IR: homeostatic model assessment for insulin resistance; HOMA-S: homeostatic model assessment for insulin sensitivity; ICAM-1: intercellular adhesion molecule-1; IGF-1: insulin-like growth factor 1; LDL-C: low-density lipoprotein cholesterol; MET: metabolic equivalent; MVPA: moderate-to-vigorous activity; PA: physical activity; PAEE: physical activity energy expenditure; RBAP4: retinoblastoma binding protein 4; SB: sedentary behaviours; SE: standard error; SOB-R: soluble leptin receptor; T2D: type 2 diabetes; TG: triglycerides; VCAM-1: vascular cell adhesion protein 1; VO2: maximal oxygen consumption; WC: waist circumference.

### Fasting glucose

31 studies analysed associations with fasting glucose [14, 25–27, 30, 33–36, 38, 39, 42, 43, 45, 47–57, 59, 61, 62, 65, 67, 68].

#### Linear regression

11 studies found no association between sedentary behaviour and fasting plasma glucose after adjusting for MVPA [25, 26, 34, 47, 50–52, 55, 62, 65, 68]. Healy et al. [49] found a 1% increase in glucose for every additional 2 hours/day spent sitting, independently of MVPA. Van der Velde et al. [30] reported a β = -0.058 (p-value = 0.04) in the model adjusted for MVPA.

#### Linea regression by quartiles

Eight studies found no differences between quartiles of SB [35, 43, 48, 53, 54, 57, 59, 61].

Gennuso et al. [45] found associations with glucose for every hour/day increase in SB, but only for the top quartile of MVPA, which included participants accumulating ≥ 300 minutes of MVPA per week: each hour of SB increased the odds of high glucose (defined as ≥ 5.55 mmol/L) by 13%.

#### Isotemporal substitution

Three studies using substitution analyses found no associations with SB [27, 38, 67]. On the other hand, three other studies found significant results: Ekblom-Bak et al. [39] found that substituting 30 minutes of SB with 30 minutes of MVPA was associated with a 0.9% improvement in fasting glucose; substituting with LIPA did not produce significant results. Buman et al. [33] reported a 1.3% reduction in fasting glucose when substituting 30 minutes of SB with MVPA; no reduction was observed when substituting with LIPA. Healy et al. [49] reported a 2% reduction when reallocating 2 hours/day of sitting time with standing time; however, reallocations with stepping were not significant.

#### Compositional transformation

With the compositional transformation, McGregor et al. [56] found an association with MVPA (β = -0.021, p-value = 0.019), indicating a beneficial effect on glucose when MVPA is increased while reducing time spent in other behaviours. Farrahi et al. [42] also found beneficial associations for increases in MVPA (β = -0.01, p-value = 0.002) and LIPA (β = -0.03, p-value = 0.001) with compositional analysis; no significant associations were found for SB.

Chastin et al. [14] and Debache et al. [36] analysed activity substitutions within a compositional framework but found no evidence of significant reallocations.

### 2-h glucose

11 studies analysed associations with 2-h glucose [25, 26, 37, 42, 43, 47–49, 55, 57, 61].

#### Linear regression

Three studies using linear regression [25, 26, 49] did not find significant associations with SB while two studies found significant associations between sedentary behaviour and 2-h glucose after adjusting for MVPA.

Healy et al. [47] found an increase in 2-h glucose for every extra hour of SB (β = 0.23, p = 0.019); Maher et al. [55] also found that higher SB was associated with higher 2-h glucose, but with a smaller effect size (β = 0.02, p<0.05).

#### Linear regression by quartiles

Qi et al. [61] found a significant difference between quartiles of SB, with a difference between bottom and top quartiles (9.9 and 13.7 hours/day respectively) of 8mg/dL (p-value < 0.0001). Diaz et al. [37] reported significant differences between quartiles, with a difference of 11.2 mg/dL between the top and bottom quartiles. Farrahi et al. [43] did not find significant differences between quartiles of sedentary bouts accumulations after adjusting for MVPA. Mossavar-Rahmani et al. [57] did not find significant differences between quartile of SB (cut-offs of 10.8, 12, 13 hours/day) over 6 years of follow-up. Healy et al. [48] also did not find significant differences between quartiles of SB.

#### Isotemporal substitution

Healy et al. [49] found that reallocating 2 hours of sitting to standing did not produce significant benefits, while the reallocation to stepping lead to a 12% reduction in 2-h glucose (p-value < 0.001) and the reallocation from standing to stepping lead to an 11% reduction (p-value = 0.005).

#### Compositional transformation

With a compositional paradigm, Farrahi et al. [42] found significant associations between SB and 2-h glucose however only for participants accumulating less than 7.5 hours/day of sleep; MVPA was beneficially associated to glucose regardless of sleep time (β_**<7.5h/d**_ = -0.06, p-value < 0.001, β_**>7.5h/d**_ = -0.04, p-value = 0.04), while no significant associations were reported for LIPA.

### Fasting insulin

19 studies analysed on fasting insulin [14, 25–27, 33–35, 39, 42, 43, 48, 53–57, 59, 61, 68].

#### Linear regression

Two studies using linear models did not find significant associations with SB [26, 68].

Carson et al. [34] found a small but significant positive association with SB (β = 0.022 pmol/L), and Barone Gibbs et al. [25] found that each extra hour of SB was associated with a 4.8% increase of insulin. Maher et al. [55] found a significant association with β = 0.08 for the model adjusted for MVPA.

#### Linear regression by quartiles

Celis-Morales et al. [35] reported mean values (SD) per quartiles of SB (cut-points: 7.45, 8.72, 9.6 hours/day) with differences being significant (Q1: 2.82 (1.22), Q2: 6.1 (1.26), Q3: 9.11 (1.22), Q4: 15.9 (1.29) mU/L, p-value = 0.0001). Loprinzi et al. [53] divided groups into active and not sedentary (G1, > 150 minutes/week of MVPA and LIPA > SB), active and sedentary (G2, > 150 minutes/week of MVPA and LIPA < SB), inactive and not sedentary (G3, < 150 minutes/week of MVPA and LIPA > SB) and inactive and sedentary (G4, < 150 minutes/week of MVPA and LIPA < SB) and found that compared to G4, G1 and G3 had reduced insulin (by a factor of 2.47 and 1.74 respectively), but not G2. Lynch et al. [54] found significant associations only for SB greater than 9.84 hours/day, but the difference between quartiles was still significant (p-value = 0.01), with a difference of 12 pmol/L between top (9.84 hours/day) and bottom quartiles (7.74 hours/day); however, after adjusting for waist circumference, only the difference between the first and third quartile remained significant. Healy et al. [48] reported a significant difference of 11.6 pmol/L between top and bottom quartiles of SB, which differed by 2.3 hours of sedentary time. Peterson et al. [59] found a significant difference between high, low and moderate SB groups, but only for the high MVPA subgroup. Qi et al. [61] reported significant differences between quartiles of SB, with the most sedentary quartile having fasting insulin increased by 1.3 mU/L compared to the least sedentary quartile. Farrahi et al. [43] found significant differences between couch potatoes (highest number of sedentary bouts interrupted less frequently) and short sitters (accumulating SB in short bouts) with a % difference of -8.8 (p-value < 0.001); no significant difference were found between couch potatoes, prolonged sitters (accumulating SB in bouts > 15–30 minutes) and breakers (accumulating less SB, which was frequently interrupted by longer non-SB time).

Only one study [57] did not find significant differences between quartiles of SB.

#### Isotemporal substitution

Buman et al. [33] found that substituting 30 minutes of SB to LIPA reduced the risk ratio to 0.99 and caused a reduction in insulin of 2.4%; substituting with MVPA reduced the RR to 0.87 and caused a reduction in insulin of 14.5%.; moreover, reallocating 3 hours/day of sedentary time to LIPA produced the same insulin reduction as reallocating 30 minutes/day of sedentary time to MVPA. Ekblom-Bak et al. [39] reported a -0.001% change about the mean when 10 minutes of SB were replaced with 10 minutes of LIPA. Whitaker et al. [27] reported a significant reduction in insulin (β = 0.2 ***μ***U/mL, p-value = 0.012) when 30 minutes/day of SB were replaced with LIPA; the reduction was greater if SB was replaced with MVPA (β = 0.73 ***μ***U/mL, p-value = 0.04) and if LIPA was replaced with MVPA (β = -0.54 ***μ***U/mL, p = 0.049).

#### Compositional transformation

Using a compositional framework, Chastin et al. [14] reported a reduction of 0.001% in insulin if 10 minutes of SB were replaced with 10 minutes of LIPA; this is also confirmed in linear regression with compositional transformation, finding an association between insulin and LIPA (β = -0.13, p-value = 0.033). Farrahi et al. [42] found significant associations, stratified by sleep duration (with cutoff 7.5 hours/day), with SB (β_**<7.5h/d**_ = 0.25, p-value = 0.001, β_**>7.5h/d**_ = 0.20, p-value = 0.036) with a compositional transformation; associations with increases in LIPA and MVPA with corresponding decreases in other behaviours were also significantly associated to fasting insulin (LIPA: β_**<7.5h/d**_ = -0.24, p-value < 0.001, β_**>7.5h/d**_ = -0.3, p-value = 0.001; MVPA: β_**<7.5h/d**_ = -0.18, p-value < 0.001, β_**>7.5h/d**_ = -0.15, p-value = 0.04). Farrahi also reported favourable reallocations between SB and physical activity, with MVPA producing more pronounced benefits.

McGregor et al. [56] reported no significant associations for SB, but found a significant association with MVPA (β = -0.116, p-value < 0.001) using a compositional paradigm, which implies reallocating other behaviours including SB for MVPA.

### 2-h insulin

Only three studies [26, 42, 43] reported on 2-h insulin.

#### Linear regression

One study analysed associations with 2-h insulin with linear regression, reporting no associations [26].

#### Linear regression by quartiles

Farrahi et al. [43] found a significant % difference of -6.5 (p-value = 0.048) between couch potatoes (highest number of sedentary bouts interrupted less frequently) and short sitters (accumulating SB in short bouts), but differences with the other sedentary groups disappeared after adjustment for MVPA.

#### Compositional transformation

Farrahi et al. [42] reported significant associations with SB (β = 0.22, p-value = 0.001), LIPA (β = -0.3, p-value < 0.001) and MVPA (β = -0.28, p-value < 0.001) within a compositional framework, and additionally they reported significant reallocations between SB and both LIPA and MVPA, with the latter yielding a higher effect size.

### HOMA-IR

15 studies analysed associations with HOMA-IR [14, 25, 26, 29, 35, 37, 39, 42, 44, 54, 57, 59, 61, 63, 68].

#### Linear regression

Four studies found no significant associations between HOMA-IR and SB [26, 44, 63, 68].

One study [25] found that each hour of SB was associated with an increase in HOMA-IR of 5.8%, which persisted after adjustments for covariates but not in the longitudinal analysis.

#### Linear regression by quartiles

Three studies found no differences between groups or quartiles of SB [37, 57, 59]. Celis-Morales et al. [35] found significant differences (p_**trend**_ = 0.0001) between quartiles of SB (Q1: 0.7 (0.27), Q2: 1.52 (0.28), Q3: 2.21 (0.27), Q4: 4.05 (0.28)); Honda et al. [29] found means of 1.19, 1.26, 1.28, 1.39 for < 6, 6–8, 8–10 and > 10 hours/day respectively. Lynch et al. [54] reported significantly lower HOMA-IR between the first and third quartiles of SB (difference of 2.19) in the fully adjusted model. Qi et al. [61] found significant differences between quartiles of SB (9.9, 11.6, 12.6, 13.7 hours/day), with a difference of 0.33 between the top and bottom quartiles.

#### Isotemporal substitution

Ekblom-Bak et al. [39] reported that substituting 30 minutes of SB with LIPA was associated with 3.1% lower HOMA-IR, and 12.4% lower if replaced with MVPA; they also found that the RR decreased linearly as increasing bout lengths were substituted.

#### Compositional transformation

The compositional analysis conducted by Farrahi et al. [42] obtained significant associations between SB and HOMA-IR only for sleep durations of < 7.5 hours/day (β = 0.26, p-value = 0.001); significant associations were found both for compositions increasing LIPA and MVPA (LIPA: β_**<7.5h/d**_ = -0.26, p-value < 0.001, β_**>7.5h/d**_ = -0.36, p-value < 0.001; MVPA: β_**<7.5h/d**_ = -0.18, p-value < 0.001, β_**>7.5h/d**_ = -0.16, p-value < 0.001).

Substitution analyses with a compositional transformation reported significant results: Chastin et al. [14] reported a -0.001% change from the mean when 10 minutes of SB were replaced with LIPA and a -0.002% change if replaced with MVPA; the results where similar to the ones obtained in compositional linear regression only for LIPA (β = -0.15, p-value = 0.02).

### Insulin sensitivity

Six studies analysed associations for insulin sensitivity [26, 32, 33, 46, 48, 55].

#### Linear regression

Two studies found no association between insulin sensitivity and SB when adjusting for MVPA [32, 46].

Maher et al. [55] reported a β = -0.08 (p-value < 0.001) for the association with SB adjusted for MVPA.

#### Linear regression by quartiles

Lahjibi et al. [26] found a significant difference of 24 ***μ***mol∙min^**-1**^∙kg_**FFM**_^**-1**^∙nmol/L^**-1**^ between the 50.4 hours/week quartile (45.9 for women) and the 62 hours/week one (57.4 for women) of SB time. Likewise, Healy et al. [48] reported significant differences between quartiles of SB: a 36% difference was observed between the bottom and top quartiles, which differed by 2.3 hours/day of sedentary time.

#### Isotemporal substitution

Buman et al. [33] found that substituting 30 minutes of SB with LIPA led to a 2.3% reduction in sensitivity and substituting with MVPA led to a 11.5% reduction.

### Diabetes incidence

Four studies reported on diabetes incidence [25, 28, 29, 66] and three found statistically significant increasing odds of diabetes for increasing SB time [25, 29, 66].

#### Linear regression

Barone Gibbs et al. [25] reported that participants spending more than 10 hours/day in SB had 3.8 times greater odds ratio of diabetes compared to participants who were sedentary for less than 6 hours/day over 7 days; each hour of SB was associated with an odds ratio increasing of 22%. Van der Velde et al. [66] found that for each 1.63 hours/day of SB, the odds ratio increased by 1.35 (CI 1.18, 1.55).

#### Linear regression by quartiles

Honda et al. [29] found that after adjusting for MVPA, only SB of more than 10 hours/day was associated with an odds ratio of diabetes (OR = 1.84, p-value = 0.04).

### HbA1c

Seven studies reported on hbA1c [25, 31, 37, 49, 50, 57, 64].

#### Linear regression

Three studies [25, 49, 64] did not find significant associations between SB and HbA1c. Barone Gibbs et al. [25] did not find cross-sectional associations with SB, but the longitudinal association almost reached significance (p-value = 0.06). Mossavar-Rahmani et al. [57] found differences between quartiles of SB, but they disappeared after adjustment for MVPA.

Honda et al. [29] found that HbA1c was significantly associated with SB (*β* = 0.009, p-value = 0.006).

#### Linear regression by quartiles

Bakrania et al. [31] compared sedentary and inactive individuals to sedentary active and non-sedentary active individuals, who had a HbA1c reduction of 0.11% (p-value = 0.009) and 0.12% (p-value = 0.003) respectively; no significant benefits in HbA1c were observed for the non-sedentary inactive group.

### CRP

15 studies analysed associations with CRP [14, 30, 34, 40, 41, 48, 53–56, 58, 59, 61, 63, 68].

#### Linear regression

Seven studies [30, 34, 40, 41, 58, 63, 68] found no significant association or quartile differences between CRP and SB.

Maher et al. [55] reported a coefficient *β* = 0.03 for the association between SB and CRP adjusted for MVPA.

#### Linear regression by quartiles

Three studies found no differences in CRP between quartiles or groups of SB [54, 59, 61].

Healy et al. [48] found a difference of 0.04 mg/dL between top and bottom quartiles of SB (the difference in SB was 2.3 hours/day). Between groups of activity (G1: active and non-sedentary, G2: active and sedentary, G3: inactive and non-sedentary, G4: inactive and sedentary), Loprinzi et al. [53] found that G1 and G2 had reduced levels of CRP compared to G4 (of -0.12 and -0.1 respectively) while G3 was not significantly different.

#### Compositional transformation

Chastin et al. [14] found a beneficial 0.001% change in the mean when 10 minutes of SB were replaced with 10 minutes of MVPA within a compositional framework. Linear regression with compositional analysis confirmed the results, with β = -0.12 (p-value < 0.001). McGregor et al. [56] also found associations between MVPA and CRP (β = -0.162, p-value = 0.005).

### IL-6

Three studies reported on IL-6 [41, 58, 60].

#### Linear regression

Elhakeem et al. [41] found no significant association with SB after adjusting for MVPA. However, Parson et al. [58] found an increase of 4.7% for every 30 minutes of SB.

#### Isotemporal substitution

Phillips et al. [60] found that replacing 30 minutes of SB with LIPA increased IL-6 of 0.34 (standardised β), and replacing SB with MVPA produced a decrease of 0.3.

## Discussion

From the combined analyses of all the studies included, no association exists between SB and fasting glucose, HbA1c or CRP; the overall quality of the studies included is medium for all three biomarkers. We found evidence of a positive association with fasting insulin, 2-h insulin, incident diabetes and IL-6—with overall medium study quality. A negative association was for insulin sensitivity in healthy adults, but the overall study quality was found to be poor. No clear association could be determined for HOMA-IR, with overall study quality being medium. Table 3 provides a summary of findings for each biomarker, stratified by analytical approach.

**Table 3:** Summary of findings table. Summarising the results from the included studies stratified by biomarker and analytical method. For the overall association, 1 means association found,? is inconclusive, 0 is no association.

	Number of studies	% studies reporting associations	Overall association	Average quality	Summary of findings
**Fasting glucose**					
Linear regression	13	15%	0	Medium	11 studies reported no associations. 1 study found a positive relationship between glucose and SB. 1 study reported a negative association.
Quartiles	9	11%	0	Medium	8 studies reported no differences between groups of SB or/and MVPA. 1 study found association with higher odds of high glucose for MVPA ≥ 300 mins/week.
ISM	6	33%	?	Medium	3 studies found no associations. 2 studies found that substituting 30 mins of SB with MVPA, but not LIPA, reduced glucose. 1 study found a significant association with sitting-standing reallocations, but not with sitting-stepping.
Compositional	4	50%	?	Medium	2 studies found no associations for compositional reallocations. 1 study found associations for increases in MVPA and LIPA (implies reduction in other activities including SB). 1 study only for increases in MVPA.
**Overall**	**31**	**19%**	**0**	**Medium**	**Combined results from linear regression and linear regression by quartiles suggest no association between SB and glucose. ISM and compositional transformation lead to inconclusive results. Overall, there seems to be no association between glucose and SB.**
**2-h glucose**					
Linear regression	5	40%	?	Medium	3 studies found no associations. 2 studies found increasing 2-h glucose for increasing SB.
Quartiles	5	40%	?	Medium	3 studies found no differences between quartiles of SB. 2 studies found that quartile with highest SB had higher 2-h glucose than quartile with lowest SB.
ISM	1	100%	1	Poor	1 study found that sitting-stepping and standing-stepping substitutions are beneficial.
Compositional	1	100%	1	Medium	1 study found positive association for increasing compositional SB only for sleep < 7.5 h/day. Increasing compositional MVPA has a negative association regardless of sleep time.
**Overall**	**11**	**50%**	**?**	**Medium**	**Combined results from linear regression and linear regression by quartiles provide inconclusive evidence. ISM and compositional transformation suggest that replacing SB with stepping and MVPA may be beneficial. Overall, there is inconclusive evidence for associations between 2-h glucose and SB.**
**Fasting insulin**					
Linear regression	5	60%	1	Medium	2 studies found no associations. 3 studies found positive associations between SB and insulin.
Quartiles	8	88%	1	Medium	1 study found no differences between quartiles of SB. 3 studies found differences between quartiles of SB and insulin (high SB = high insulin). 1 study found associations only for highest SB quartile (cut-off 9.84 h/day). 1 study found that inactive people had worse insulin if they were also sedentary. 1 study found that differences between SB groups exist only for high MVPA levels. 1 study found only difference between long-short bouts of SB.
ISM	3	100%	1	Medium	2 studies found that substituting 30 minutes MVPA is better than LIPA; equal benefits are observed If 2h LIPA or 30 mins of MVPA are substituted to SB. 1 study found small benefit of substituting SB with LIPA.
Compositional	3	67%	1	Medium	1 study found no association for compositional SB. 1 study found significant associations with compositional SB. 1 study found benefits for reallocations between SB and LIPA, and another study found that substituting with MVPA is better than LIPA.
**Overall**	**19**	**79%**	**1**	**Medium**	**Results from different analytical methods agree and there is evidence of association between fasting insulin and SB. Substituting SB with both LIPA and MVPA reduces insulin, with MVPA being more beneficial.**
**2-h insulin**					
Linear regression	1	0%	0	Medium	No significant associations.
Quartiles	1	100%	1	Poor	Significant difference between long and short bouts of SB.
ISM	0	N/A	N/A	N/A	No studies analysed the association between SB and insulin sensitivity with a compositional transformation.
Compositional	1	100%	1	Poor	Significant associations between compositional SB, LIPA and MVPA. Significant reallocations between LIPA and MVPA, with MVPA being more beneficial.
**Overall**	**3**	**67%**	**1**	**Poor**	**Linear regression provides evidence of no association with SB, while regression by groups of SB leads to evidence of association. Compositional analysis gives evidence of association. Overall, there is some evidence of association between 2-h insulin and SB, and reallocating SB to LIPA and MVPA could be beneficial.**
**HOMA-IR**					
Linear regression	5	20%	0	Medium	4 studies found no significant associations with SB. 1 study found positive associations in the cross-sectional analysis but not in the longitudinal.
Quartiles	7	57%	?	Medium	3 studies found no differences between quartiles of SB. 4 studies found significant differences between quartiles of SB.
ISM	1	100%	1	Poor	1 study found significant reallocations between LIPA and MVPA, with MVPA being more beneficial.
Compositional	2	100%	1	Medium	1 study found significant association with SB for sleep < 7.5 h/day. 1 study found compositional LIPA associated to HOMA-IR and substituting SB with both LIPA and MVPA was beneficial, with MVPA being more beneficial.
**Overall**	**15**	**53%**	**?**	**Medium**	**Linear regression provides evidence of no association between SB and HOM-IR, while regression by quartiles leads to inconclusive evidence. ISM and compositional analysis provide evidence of an association. Given contrasting results from different analytical methods, there is inconclusive evidence for associations between HOMA-IR and SB.**
**Insulin sensitivity**					
Linear regression	3	33%	?	Medium	2 studies found no associations with SB. 1 study found a negative association between SB and insulin sensitivity.
Quartiles	2	100%	1	Medium	2 studies found significant differences between quartiles of SB.
ISM	1	100%	1	Medium	1 study found significant reallocations between LIPA and MVPA, with MVPA being more beneficial.
Compositional	0	N/A	N/A	N/A	No studies analysed the association between SB and insulin sensitivity with a compositional transformation.
**Overall**	**6**	**67%**	**1**	**Medium**	**Studies using linear regression provide inconclusive evidence of association, while regression by quartiles and ISM provide evidence of an association between insulin sensitivity and SB.**
**Diabetes**					
Linear regression	2	100%	1	Medium	2 studies found significant associations between SB and incident diabetes
Quartiles	2	50%	?	Medium	1 study found no differences between tertiles of SB. 1 study found associations with SB only for SB > 10 h/day.
ISM	0	N/A	N/A	N/A	No studies analysed the association between SB and incident diabetes with a isotemporal substitution analysis.
Compositional	0	N/A	N/A	N/A	No studies analysed the association between SB and insulin sensitivity with a compositional transformation.
**Overall**	**4**	**75%**	**1**	**Medium**	**Studies using linear regression provide evidence of association, while regression by quartiles gives inconclusive evidence. Overall, there is evidence of an association between SB and incident diabetes.**
**HbA1c**					
Linear regression	4	25%	0	Medium	3 studies found no significant associations between SB and hbA1c. 1 study found a small positive association.
Quartiles	3	33%	?	Medium	2 studies found no differences between quartiles of SB and hbA1c. 1 study found differences between groups split by SB and MVPA (high VS low).
ISM	0	N/A	N/A	N/A	No studies analysed the association between SB and hbA1c with a isotemporal substitution analysis.
Compositional	0	N/A	N/A	N/A	No studies analysed the association between SB and hbA1c with a compositional transformation.
**Overall**	**7**	**29%**	**0**	**Medium**	**Overall, there is no evidence of an association between hbA1c and SB.**
**CRP**					
Linear regression	8	13%	0	Medium	7 studies reported no significant associations with SB. 1 study found a positive association between SB and CRP.
Quartiles	5	40%	?	Medium	3 studies found no differences between quartiles of SB. 1 study found differences in CRP between SB quartiles. 1 study found that active not sedentary and active sedentary groups had lower CRP than inactive sedentary.
ISM	0	N/A	N/A	N/A	No studies analysed the association between SB and CRP with a isotemporal substitution analysis.
Compositional	1	50%	?	Medium	1 study reported no significant association with compositional SB, but only with MVPA. 1 study found a significant association with compositional SB and 10-minute substitutions with MVPA.
**Overall**	**15**	**27%**	**0**	**Medium**	**Linear regression providence evidence of no association between SB and CRP, while regression by SB groups give and compositional analysis inconclusive results. Overall, the evidence is inconclusive.**
**IL-6**					
Linear regression	2	50%	?	Medium	1 study did not found associations with SB, while another one did.
Quartiles	0	N/A	N/A	N/A	No studies analysed the association between SB and IL-6 by groups of SB.
ISM	1	100%	1	Medium	1 study reported that replacing SB with LIPA increases IL-6, while replacing with MVPA decreases it.
Compositional	0	N/A	N/A	N/A	No studies analysed the association between SB and IL-6 with a compositional transformation.
**Overall**	**3**	**67%**	**1**	**Medium**	**Linear regression provided inconclusive evidence on the association with SB, while ISM provided evidence of beneficial reallocations. Overall, there is some evidence of the association between IL-6 and SB.**

CRP: C-reactive protein; hbA1c: glycated haemoglobin; HOMA-IR: homeostasis model assessment for insulin resistance; IL-6: interleukin 6; ISM: isotemporal substitution model; LIPA: low-intensity physical activity; MVPA: moderate-to-vigorous physical activity; SB: sedentary behaviours.

The present finding that fasting insulin is associated with SB is consistent with a previous review by Powell et al. [19], though they reported associations between SB and fasting glucose, which is not found in this review. This difference may be due to the use of unadjusted data in their meta-analysis, as in the present review we found that the negative association between SB and fasting glucose disappeared when models were adjusted for MVPA. Brocklebank et al. [2] reported a negative association with insulin sensitivity—also found in this review—but insufficient evidence for other biomarkers of T2D. This difference may be due to the lower number of studies included in the previous review, to the inclusion only of populations with diabetes and/or at high risk of metabolic conditions and to the lack of studies using compositional or isotemporal substitution analyses, which reported significant associations in the present review. A review on elderly individuals [3] reported similar findings to the present study: SB was negatively associated with fasting insulin and HOMA-IR, while no association was found with glucose levels.

Studies using the ISM or a compositional transformation report significant associations between T2D markers and reallocations between SB and PA. Benefits were reported when SB was substituted with either LIPA or MVPA for 2-h glucose, fasting insulin, 2-h insulin, HOMA-IR, insulin sensitivity, CRP and IL-6. However, for 2-h glucose and HOMA-IR, the overall quality for studies using ISM was poor and therefore, we cannot exclude the presence of bias in the composite result. For incident diabetes and hbA1c, none of the included studies employed ISM or a compositional transformation to analyse potential associations. For insulin, substituting SB with LIPA resulted in a lower reduction in RR—albeit still significant—than for MVPA substitutions. Other reviews analysing the benefits of reallocating SB with PA found reductions in cardiometabolic biomarkers [15, 16] for substitutions of both LIPA and MVPA. In children, only MVPA reallocations seem to be associated to a reduction in body fat, with greatest benefits for 60-minute reallocations, while substitutions for LIPA do not have evidence of association [17]. A review including only compositional associations with biomarkers of glucose and insulin control [18] did not find conclusive evidence of association with measures of SB and PA. Despite this disagreements in the evidence obtained through ISM and compositional analysis, this review observed evidence of agreement between the two methods, as also speculated by a recent consensus on methodology to analyse associations with accelerometer-measured PA [69].

On the other hand, the results derived from standard linear regression, from regression stratified by SB levels, from ISM and from compositional approaches are in disagreement for fasting glucose, 2-h glucose, 2-h insulin, insulin sensitivity, HOMA-IR, incident diabetes, CRP and IL-6. The absence of associations between SB and T2D markers when standard regression is employed, indicates that the assumption of linearity between exposure and outcomes may be wrong. The studies included in this review that analysed the participants by quartiles report a dose-response relationship between SB and biomarkers and independent associations with SB only for high duration of sedentary time: Lynch et al. [54] reported significant associations between SB, fasting insulin and HOMA-R for more than 8.8 hours/day of SB; Healy et al. [48] found a cut-off of 7.24 hours/day of SB that increase fasting insulin; Honda et al. [29] found that more than 10 hours/day of SB significantly increase the OR of insulin resistance and diabetes. A meta-analysis [70] found evidence of a logarithmic dose response relationship between SB and all-cause mortality and an accelerometer-measured 9-hour cut-off of SB—adjusted for MVPA—after which hazard ratios of all-cause mortality increased. For studies measuring SB with subjective methods, the cut-off was around 7 hours; a similar cut-off (6–8 hours) for subjective SB was also found by Patterson et al. [71]. The discrepancy between cut-offs found with device-measured and self-reported measures of SB are likely due to participants underestimating the time spent in SB: a recent meta-analysis quantified the difference between device-measured and self-reported measures SB as ~1.74 hours/day [72]. As such, a threshold between 7–9 hours/day of SB may be significant not only for all-cause and CVD mortality, but also for individual biomarkers of T2D. However, due to the scarce evidence found by this review, it is not possible to recommend a specific cut-off for SB that significantly increases biomarkers of T2D. Despite the evidence of deleterious effects of high SB, a review on the interaction between subjectively measured MVPA and SB [20] found that adults who engaged in MVPA for 60–75 minutes/day did not have an increase in mortality risk even for sitting times greater than 8 hours/day. However, the present finding that biomarkers of T2D are associated to SB for many hours per day suggests that such levels of MVPA are not achieved by the participants in the selected studies and are likely unattainable by a large part of the population. As a result, displacing SB with LIPA, or a mixture of MVPA and LIPA, could be the most feasible option. Supporting the case for displacing SB with longer bouts of LIPA rather than shorter MVPA bouts, a randomised crossover trial [73] found that substituting one hour of daily sitting with MVPA (cycling) does not improve insulin nor glucose, while replacing 6 hours/day of sitting with 4 hours of leisurely walking and 2 hours of standing has significant beneficial effects on both insulin and glucose.

### Implications of findings

The present findings support the case for time-based guidelines to reduce prolonged periods of sedentary time in the 18–64 healthy population. Several studies included in the review reported clinically significant changes in biomarkers. For example, Barone Gibbs et al. [25] reported a 4.8% increase in fasting insulin for every additional hour of SB; Celis-Morales et al. [35] found that the highest SB quartile has a 6.2% increase in insulin compared to the lowest quartile of SB. According to Buman et al. [33], substituting 30 minutes of SB with LIPA reduced insulin by 2.4%, and substituting with MVPA reduced it by 14.5%. These effect sizes are comparable to the effect of a LIPA intervention [74], which produced a 18.2% reduction after three months in high-risk patients for T2D. Additionally, a 20% difference in area under the insulin response curve, was found to be associated with a 10% difference in coronary mortality risk [75], thus suggesting that SB reallocations could have a clinically meaningful effect. However, other studies included in the review reported much smaller percent reductions in insulin: Carson et al. [34] reported a 0.03% change for every additional hour of SB; Healy et al. [48] and Lynch et al. [54] found 0.32% and 0.34% insulin difference between top and bottom quartiles of SB (difference in SB of 2.3 and 2.1 h/day), respectively. This potentially invalidates the claim of clinical relevance, and a meta-analysis to calculate a pooled effect size is needed to formulate a reliable conclusion.

Despite finding some evidence of a cut-off between 7–9 hours/day of SB, more evidence is required to support this value specifically for T2D biomarkers. Moreover, this review highlights the need for more evidence on how to spend the time gained from the reduction of SB most effectively. Studies analysing associations by quartiles found evidence on the deleterious effects of SB—especially for long sedentary periods—but, unlike other analytical approaches, cannot inform on how to combine PA of different kinds and intensity to benefit health. Current PA guidelines focus on moderate and vigorous PA, but lower intensity activities can also benefit T2D markers, as found in this review. More prolonged but less intense exercise may be easier for the elderly or impaired, while short but more intense activities could be as beneficial for those who cannot engage in prolonged sessions. Future research should analyse the effects of different bout lengths on a wide array of metabolic biomarkers to provide the data necessary to develop new and quantified guidelines on SB and LIPA, alongside the already present MVPA recommendations.

Additionally, future studies should consider using analytical approaches that examine the interaction between SB and PA of various intensities. While ISM allow to evaluate the association with reallocations of SB with PA, it does not allow to examine time compositions including different kinds of PA, as the reallocation is done between SB and one behaviour at a time. Moreover, this method presents the same limitations as standard linear regression, i.e. multicollinearity and assumption of linearity, and therefore is not recommended [69]. Other approaches, such as compositional transformation, may be better suited to assess the joint effect of SB and PA on measures of health. For example, Debache et al. [36] analysed different combinations of daily behaviours (lying, sitting, standing, LIPA and MVPA) with compositional models and reported varying differences in cardiometabolic biomarkers from the sample mean.

### Strengths and limitations

While this review found evidence of the benefits of different PA intensities (LIPA and MVPA), it was not possible to systematically assess how bout duration affects T2D biomarkers, as all studies but two only reallocated for 30 minutes of activity. Chastin et al. [14] found a minimal reduction in glucose, insulin and HOMA-IR for a 10-minute reallocation; but given that the results were reported as % change about the mean, it was not possible to compare their results to the studies substituting for 30-minute bouts which used risk ratios. Healy et al. [49] reallocated 120 minutes of SB to LIPA and MVPA and found significant but small, reductions in fasting and 2h-glucose, suggesting that longer substitutions may influence glucose, which is not seen for shorter time substitutions. Therefore, more studies are needed to assess the impact of substituting different bout lengths. Moreover, a greater variety of activities should also be included in the analyses, as different types of activities have different effects on T2D markers [76]. Additionally, this review raises the issue around the discrepancy between results obtained with linear regression and other analytical methods. Finally, a major limitation of the present review is the lack of a pre-registered protocol. However, despite the absence of a review protocol, we adhered to the best practices for conducting a systematic review in order to minimise the potential for bias.

This review has several strengths: it solely includes device-measured methods for monitoring activity (specifically by accelerometer) and studies that adjusted for MVPA in the analyses. Additionally, only studies examining associations in healthy adults (18–64) were included, to consider evidence relevant to the current age-specific guidelines. To the authors’ knowledge, this is the first review including both results from standard and stratified linear regression and compositional approaches (including ISM) whilst also comparing the methods. Despite other analytical approaches exist for analysing the association between accelerometer data and health outcomes, such as functional analysis [77] and multivariate pattern analysis [78], we were unable to include them in the present review, as studies employing such methods did not fulfil the selection criteria. This review presents other limitations such as the lack of prospective studies and randomised controlled studies, which are lacking in the literature; consequently, no considerations around causality can be made at this time. We could not answer the question about how different types of activity affect marker levels for several of the included outcomes, as few studies reported on reallocations with LIPA. Equally, we could not find evidence for how substitutions with different bout durations impact the risk reduction magnitude, as most studies reallocated only 30 minutes between activities, and reallocated only one kind of activity at a time. No meta-analysis could be done given the high heterogeneity of studies in terms of devices and activity cut-offs used, effect sizes and statistical models.

## Conclusion

This review found evidence of the negative association between SB and fasting 2-h glucose, fasting insulin, 2-h insulin, incident diabetes and IL-6. In addition, we found some evidence of a threshold of 9 hours/day, after which the effect of SB on T2D biomarkers is independent of MPVA, likely due to failing to accumulate enough MVPA to counteract the increased risk. However, the evidence is too scarce to provide definite recommendations regarding a time-specific cut-off for SB. While this review provides evidence of the health benefits associated with LIPA and MVPA, it was not possible to determine what durations and compositions are required to compensate for the reduction in intensity. Further work to confirm a time-based threshold and enable quantitative recommendations for SB, as well as flexible and achievable replacement of SB time is needed to provide actionable evidence for policy makers and clinicians.

## Supporting information

S1 File (DOCX)
