# Supplementary material for: The association between sedentary behaviour, physical activity and type 2 diabetes markers: A systematic review of mixed analytic approaches
Source: PLoS One. 2022 May 11;17(5):e0268289. doi: 10.1371/journal.pone.0268289 (PMC9094551; doi:10.1371/journal.pone.0268289)
Supplement: S1 File — (DOCX) [file pone.0268289.s001.docx]

Supporting information

# S1 Text. Ovid and Embase database search terms

1. exp Exercise/

2. life style/ or healthy lifestyle/ or life change events/

3. exp Sports/

4. exp Exercise Therapy/

5. exp Physical Fitness/

6. exercis*.ti,ab.

7. Physical activit*.ti,ab.

8. motor activit*.ti,ab.

9. physical training.ti,ab.

10. leisure activit*.ti,ab.

11. resistance training.ti,ab.

12. ((lifestyle or life style) adj3 (intervention? or change* or modif* or program or programme)).ti,ab.

13. sport*.ti,ab.

14. 1 or 2 or 3 or 4 or 5 or 6 or 7 or 8 or 9 or 10 or 11 or 12 or 13

15. exp Sedentary Behavior/

16. screen time/

17. sedentar*.ti,ab.

18. (sedentar* adj3 (behavio* or time*)).ti,ab.

19. (time* adj3 sedentar*).ti,ab.

20. (sedentar* adj3 lifestyle*).ti,ab.

21. ((break* or interrupt*) adj3 sedentar* adj3 time*).ti,ab.

22. (sitting adj3 time*).ti,ab.

23. sit*.ti,ab.

24. stationary behavio?r.ti,ab.

25. stand*.ti,ab.

26. lying.ti,ab.

27. bout*.ti,ab.

28. television viewing.ti,ab.

29. computer viewing.ti,ab.

30. television game*.ti,ab.

31. computer game*.ti,ab.

32. video game*.ti,ab.

33. low energy expenditure.ti,ab.

34. screen based entertainment.ti,ab.

35. screen-based entertainment.ti,ab.

36. screen time.ti,ab.

37. 15 or 16 or 17 or 18 or 19 or 20 or 21 or 22 or 23 or 24 or 25 or 26 or 27 or 28 or 29 or 30 or 31 or 32 or 33 or 34 or 35 or 36

38. 14 and 37

39. exp Accelerometry/

40. (objective* adj3 (evidence or data or measure* or assess*)).ti,ab.

41. (measure* adj3 objective*).ti,ab.

42. acceleromet*.ti,ab.

43. (acceleromet* adj3 (measure* or assess*)).ti,ab.

44. actigraph*.ti,ab.

45. pedomet*.ti,ab.

46. inclinomet*.ti,ab.

47. (activ* adj3 monitor*).ti,ab.

48. 39 or 40 or 41 or 42 or 43 or 44 or 45 or 46 or 47

49. 38 and 48

50. insulin/ or insulin, regular, human/

51. insulin resistance/ or metabolic syndrome/

52. Glucose Intolerance/

53. Diabetes Mellitus, Type 2/

54. exp Glucose/

55. Hyperglycemia/

56. ((impaired fasting adj2 glucose) or IFG or impaired FPG).ti,ab.

57. ((impaired glucose adj (tolerance or metabolism)) or IGT).ti,ab.

58. ((blood or plasma) adj3 glucose).ti,ab.

59. (glucose adj3 metabol*).ti,ab.

60. glucose.ti,ab.

61. insulin.ti,ab.

62. (insulin adj3 (sensitiv* or resistan*)).ti,ab.

63. (homeostasis adj3 model adj3 assessment).ti,ab.

64. ("HbA(1c)" or HbA1 or HbA1c or "HbA 1c" or ((glycosylated or glycated) adj h?emoglobin)).ti,ab.

65. (inflammat* adj3 (marker* or biomarker*)).ti,ab.

66. (C-reactive adj3 protein*).ti,ab.

67. (Interleukin 6 adj3 protein*).ti,ab.

68. (risk adj3 (type 2 or type II or diabetes or T2D* or NIDDM)).ti,ab.

69. (type 2 adj3 diab*).ti,ab.

70. (metabol* adj3 risk adj3 factor*).ti,ab.

71. (marker* adj3 metabol*).ti,ab.

72. (risk adj3 factor* adj3 metabol*).ti,ab.

73. 50 or 51 or 52 or 53 or 54 or 55 or 56 or 57 or 58 or 59 or 60 or 61 or 62 or 63 or 64 or 65 or 66 or 67 or 68 or 69 or 70 or 71 or 72

74. 49 and 73

# S2 Text. Cochrane Library database search

1. Prediabetic state/

2. Glucose Intolerance/

3. (prediabet* or pre diabet*).tw.

4. intermediate hyperglyc?emi*.tw.

5. ((impaired fasting adj2 glucose) or IFG or impaired FPG).tw.

6. glucose intolerance.tw.

7. ((impaired glucose adj (tolerance or metabolism)) or IGT).tw.

8. ("HbA(1c)" or HbA1 or HbA1c or "HbA 1c" or ((glycosylated or glycated) adj h?emoglobin)).tw.

9. (risk adj3 ("type 2" or "type II" or diabetes or T2D* or NIDDM)).tw.

10. *Diabetes mellitus/pc

11. *Diabetes mellitus, Type 2/pc

12. or/1‐11

13. Life Style/

14. exp Exercise/

15. exp Exercise Therapy/

16. exp Diet/

17. exp Diet Therapy/

18. ((lifestyle or life style) adj3 (intervention? or change* or modif* or program or programme)).tw.

19. diet*.tw.

20. (nutrition* adj3 (intervention? or change* or modif* or program or programme)).tw.

21. exercis*.tw.

22. physical activit*.tw.

*23.* resistance training.tw.

24. or/13‐23

25. 12 and 24

26. (diabetes prevention adj (program* or stud* or trial?)).tw.

27. 25 or 26

28. complication?.tw.

29. mortality.tw.

30. (CHD or CVD).tw.

31. (coronary adj2 disease).tw.

32. (coronar* adj (event? or syndrome?)).tw.

33. (heart adj (failure or disease? or attack? or infarct*)).tw.

34. (myocardial adj (infarct* or isch?emi*)).tw.

35. cardiac failure.tw.

36. angina.tw.

37. revasculari*.tw.

38. (stroke or strokes).tw.

39. cerebrovascular.tw.

40. ((brain* or cerebr*) adj (infarct* or isch?emi*)).tw.

41. apoplexy.tw.

42. ((vascular or peripheral arter*) adj disease?).tw.

43. cardiovascular.tw.

44. (neuropath* or polyneuropath*).tw.

45. (retinopath* or maculopath*).tw.

46. (nephropath* or nephrotic or proteinuri* or albuminuri*).tw.

47. ((kidney or renal) adj (disease? or failure or transplant*)).tw.

48. ((chronic or endstage or end stage) adj (renal or kidney)).tw.

49. (CRD or CRF or CKF or CRF or CKD or ESKD or ESKF or ESRD or ESRF).tw.

50. (microvascular or macrovascular or ((micro or macro) adj vascular)).tw.

51. (cancer or carcino* or neoplas* or tumo?r?).tw.

52. (amputation? or ulcer* or foot or feet or wound*).tw.

53. ((risk or progress* or prevent* or inciden* or conversion or develop* or delay*) adj4 (diabetes or T2D* or NIDDM or "type 2" or "type II")).tw.

54. or/28‐53

55. 27 and 54

56. randomized controlled trial.pt.

57. controlled clinical trial.pt.

58. randomi?ed.ab.

59. placebo.ab.

60. clinical trials as topic/

61. randomly.ab.

62. trial.ti.

63. or/56‐62

64. exp animals/ not humans/

65. 63 not 64

66. 55 and 65

67. cochrane database of systematic reviews.jn. or search*.tw. or meta analysis.pt. or medline.tw. or systematic review.tw.

68. 55 and 67

69. 66 or 68

70. (2014* or 2015* or 2016* or 2017*).dc.

71. 69 and 70

72. remove duplicates from 71

# S1 Table. PRISMA checklist

| **Section and Topic** | **Item #** | **Checklist item** | **Location where item is reported** |
| --- | --- | --- | --- |
| **TITLE** | | |  |
| Title | 1 | Identify the report as a systematic review. | Page 1 |
| **ABSTRACT** | | |  |
| Abstract | 2 | See the PRISMA 2020 for Abstracts checklist. | Page 2-3 |
| **INTRODUCTION** | | |  |
| Rationale | 3 | Describe the rationale for the review in the context of existing knowledge. | Page 3-4 |
| Objectives | 4 | Provide an explicit statement of the objective(s) or question(s) the review addresses. | Page 3-4 |
| **METHODS** | | |  |
| Eligibility criteria | 5 | Specify the inclusion and exclusion criteria for the review and how studies were grouped for the syntheses. | Page 4-5 |
| Information sources | 6 | Specify all databases, registers, websites, organisations, reference lists and other sources searched or consulted to identify studies. Specify the date when each source was last searched or consulted. | Page 4 |
| Search strategy | 7 | Present the full search strategies for all databases, registers and websites, including any filters and limits used. | Suppl. Info (S1 Text, S2 Text) |
| Selection process | 8 | Specify the methods used to decide whether a study met the inclusion criteria of the review, including how many reviewers screened each record and each report retrieved, whether they worked independently, and if applicable, details of automation tools used in the process. | Page 5 |
| Data collection process | 9 | Specify the methods used to collect data from reports, including how many reviewers collected data from each report, whether they worked independently, any processes for obtaining or confirming data from study investigators, and if applicable, details of automation tools used in the process. | Page 4-5 |
| Data items | 10a | List and define all outcomes for which data were sought. Specify whether all results that were compatible with each outcome domain in each study were sought (e.g. for all measures, time points, analyses), and if not, the methods used to decide which results to collect. | Page 4 |
|  | 10b | List and define all other variables for which data were sought (e.g. participant and intervention characteristics, funding sources). Describe any assumptions made about any missing or unclear information. | Page 6 |
| Study risk of bias assessment | 11 | Specify the methods used to assess risk of bias in the included studies, including details of the tool(s) used, how many reviewers assessed each study and whether they worked independently, and if applicable, details of automation tools used in the process. | Page 5-6 |
| Effect measures | 12 | Specify for each outcome the effect measure(s) (e.g. risk ratio, mean difference) used in the synthesis or presentation of results. | Table 2 |
| Synthesis methods | 13a | Describe the processes used to decide which studies were eligible for each synthesis (e.g. tabulating the study intervention characteristics and comparing against the planned groups for each synthesis (item #5)). | Page 4-5 |
|  | 13b | Describe any methods required to prepare the data for presentation or synthesis, such as handling of missing summary statistics, or data conversions. | N/A |
|  | 13c | Describe any methods used to tabulate or visually display results of individual studies and syntheses. | Page 4-5 |
|  | 13d | Describe any methods used to synthesize results and provide a rationale for the choice(s). If meta-analysis was performed, describe the model(s), method(s) to identify the presence and extent of statistical heterogeneity, and software package(s) used. | Page 5 |
|  | 13e | Describe any methods used to explore possible causes of heterogeneity among study results (e.g. subgroup analysis, meta-regression). | N/A |
|  | 13f | Describe any sensitivity analyses conducted to assess robustness of the synthesized results. | N/A |
| Reporting bias assessment | 14 | Describe any methods used to assess risk of bias due to missing results in a synthesis (arising from reporting biases). | N/A |
| Certainty assessment | 15 | Describe any methods used to assess certainty (or confidence) in the body of evidence for an outcome. | Page 5-6 |
| **RESULTS** | | |  |
| Study selection | 16a | Describe the results of the search and selection process, from the number of records identified in the search to the number of studies included in the review, ideally using a flow diagram. | Fig1 and page 6-7 |
|  | 16b | Cite studies that might appear to meet the inclusion criteria, but which were excluded, and explain why they were excluded. | Fig1 and Table S2 |
| Study characteristics | 17 | Cite each included study and present its characteristics. | Table 2 |
| Risk of bias in studies | 18 | Present assessments of risk of bias for each included study. | Table S3 |
| Results of individual studies | 19 | For all outcomes, present, for each study: (a) summary statistics for each group (where appropriate) and (b) an effect estimate and its precision (e.g. confidence/credible interval), ideally using structured tables or plots. | 19-28. Table 3 |
| Results of syntheses | 20a | For each synthesis, briefly summarise the characteristics and risk of bias among contributing studies. | N/A |
|  | 20b | Present results of all statistical syntheses conducted. If meta-analysis was done, present for each the summary estimate and its precision (e.g. confidence/credible interval) and measures of statistical heterogeneity. If comparing groups, describe the direction of the effect. | N/A |
|  | 20c | Present results of all investigations of possible causes of heterogeneity among study results. | N/A |
|  | 20d | Present results of all sensitivity analyses conducted to assess the robustness of the synthesized results. | N/A |
| Reporting biases | 21 | Present assessments of risk of bias due to missing results (arising from reporting biases) for each synthesis assessed. | N/A |
| Certainty of evidence | 22 | Present assessments of certainty (or confidence) in the body of evidence for each outcome assessed. | N/A |
| **DISCUSSION** | | |  |
| Discussion | 23a | Provide a general interpretation of the results in the context of other evidence. | Page 33-35 |
|  | 23b | Discuss any limitations of the evidence included in the review. | Page 37-38 |
|  | 23c | Discuss any limitations of the review processes used. | Page 37-38 |
|  | 23d | Discuss implications of the results for practice, policy, and future research. | Page 35-37 |
| **OTHER INFORMATION** | | |  |
| Registration and protocol | 24a | Provide registration information for the review, including register name and registration number, or state that the review was not registered. | The review was not registered |
|  | 24b | Indicate where the review protocol can be accessed, or state that a protocol was not prepared. | Protocol was not prepared |
|  | 24c | Describe and explain any amendments to information provided at registration or in the protocol. | N/A |
| Support | 25 | Describe sources of financial or non-financial support for the review, and the role of the funders or sponsors in the review. | Done at submission |
| Competing interests | 26 | Declare any competing interests of review authors. | Done at submission |
| Availability of data, code and other materials | 27 | Report which of the following are publicly available and where they can be found: template data collection forms; data extracted from included studies; data used for all analyses; analytic code; any other materials used in the review. | N/A |

# S2 Table. List of studies excluded in full text review with reasons.

| Study | Doi | Reasons for exclusion |
| --- | --- | --- |
| Aadahl (2012) | http://dx.doi.org/10.1016/j.jsams.2012.11.137 | Wrong activity measurement (subjective or non-accelerometer) |
| Aadland (2018) | http://dx.doi.org/10.1186/s12966-018-0707-z | Paedriatic population (<18) |
| Abdullah (2015) | http://dx.doi.org/10.3923/jms.2015.105.109 | Wrong activity measurement (subjective or non-accelerometer) |
| AcostaManzano (2018) | http://dx.doi.org/10.1016/j.maturitas.2018.11.015 | Wrong exposures (SB not reported, SB not adjusted for PA, SB as inactivity, etc.) |
| Acosta-Manzano (2019) | http://dx.doi.org/10.1016/j.maturitas.2018.11.015 | Wrong outcomes |
| Adam (2013) | http://dx.doi.org/10.2337/db13-680-858 | Abstract only |
| Adams (2013) | https://dx.doi.org/10.3389/fpubh.2013.00045 | Population chosen with high risk of type 2 diabetes |
| Adams (2015) | http://dx.doi.org/10.1016/j.ypmed.2015.05.010 | Wrong exposures (SB not reported, SB not adjusted for PA, SB as inactivity, etc.) |
| Afaq (2019) | http://dx.doi.org/10.1371/journal.pone.0216354 | Wrong exposures (SB not reported, SB not adjusted for PA, SB as inactivity, etc.) |
| Alasagheirin (2018) | http://dx.doi.org/10.1111/phn.12386 | Paedriatic population (<18) |
| Alderete (2012) | https://www.asep.org/asep/asep/JEPonlineApril2012Tanya_Alderete.pdf | Paedriatic population (<18) |
| Alexandre (2017) | https://doi.org/10.2337/db17-664-889 | Population chosen with type 2 diabetes at baseline |
| Alghafri (2018) | http://dx.doi.org/10.1136/bmjdrc-2018-000605 | Population chosen with type 2 diabetes at baseline |
| Alkahtani (2015) | http://dx.doi.org/10.1186/s12889-015-2578-4 | Wrong exposures (SB not reported, SB not adjusted for PA, SB as inactivity, etc.) |
| Alkahtani (2016) | http://dx.doi.org/10.1111/obr.12401 | BMI > 30 |
| Altenburg (2015) | http://dx.doi.org/10.1016/j.ypmed.2014.12.015 | Paedriatic population (<18) |
| Alves (2016) | http://dx.doi.org/10.1055/s-0036-1584582 | Wrong activity measurement (subjective or non-accelerometer) |
| Amadid (2016) | http://dx.doi.org/10.1007/s00125-016-4046-9 | Abstract only |
| Amadid (2017) | https://doi.org/10.2337/db17-664-889 | Population chosen with type 2 diabetes at baseline |
| Amadid (2017) | http://dx.doi.org/10.1249/MSS.0000000000001362 | Population chosen with high risk of type 2 diabetes |
| Andersen (2015) | http://dx.doi.org/10.1249/MSS.0000000000000460 | Population chosen with high risk of type 2 diabetes |
| Aparicio (2018) | http://dx.doi.org/10.1007/s13105-018-0656-7 | Abstract only |
| Arvidsson (2015) | http://dx.doi.org/10.1123/jpah.2013-0222 | Wrong exposures (SB not reported, SB not adjusted for PA, SB as inactivity, etc.) |
| Assah (2009) | http://dx.doi.org/10.2337/dc08-1538 | Wrong activity measurement (subjective or non-accelerometer) |
| Atkin (2013) | http://dx.doi.org/10.1249/MSS.0b013e318282190e | Paedriatic population (<18) |
| Ayabe (2011) | http://dx.doi.org/10.1038/oby.2011.225 | Wrong exposures (SB not reported, SB not adjusted for PA, SB as inactivity, etc.) |
| Ayabe (2012) | http://dx.doi.org/10.1007/s00421-012-2342-8 | Wrong exposures (SB not reported, SB not adjusted for PA, SB as inactivity, etc.) |
| Ayala (2016) | http://dx.doi.org/10.3390/ijerph13121227 | Paedriatic population (<18) |
| Bailey (2012) | http://dx.doi.org/10.1007/s00431-012-1719-3 | Paedriatic population (<18) |
| Bailey (2013) | http://dx.doi.org/10.1371/journal.pone.0057101 | Paedriatic population (<18) |
| Bailey (2017) | http://dx.doi.org/10.1080/02640414.2016.1260150 | Paedriatic population (<18) |
| Bankoski (2011) | http://dx.doi.org/10.2337/dc10-0987 | Wrong outcomes |
| Barker (2018) | http://dx.doi.org/10.1016/j.ijcard.2017.11.080 | Paedriatic population (<18) |
| Barlow (2016) | http://dx.doi.org/10.5888/pcd13.160263 | Wrong activity measurement (subjective or non-accelerometer) |
| Baron (2014) | https://doi.org/10.1016/j.sleep.2009.02.010 | Abstract only |
| Barrett (2018) | http://dx.doi.org/10.1186/s12889-018-6064-7 | Population with underlying health conditions |
| Belcher (2015) | http://dx.doi.org/10.1123/jpah.2013-0193 | Paedriatic population (<18) |
| Bell (2010) | http://dx.doi.org/10.1123/jpah.7.2.203 | Wrong study design (not observational) |
| Bellettiere (2019) | http://dx.doi.org/10.1093/gerona/gly101 | Geriatric population (>65) |
| Benedetti (2018) | http://dx.doi.org/10.1111/dme.23_13571 | Abstract only |
| Biddle (2015) | http://dx.doi.org/10.1371/journal.pone.0143398 | Population chosen with high risk of type 2 diabetes |
| Biddle (2018) | https://dx.doi.org/10.3390/ijerph15102280 | Population chosen with high risk of type 2 diabetes |
| Biger (2010) | http://dx.doi.org/10.1111/j.1472-8206.2010.00819.x | Population with underlying health conditions |
| Bjornholt (2003) | http://dx.doi.org/10.1097/00004872-200307000-00029 | Wrong exposures (SB not reported, SB not adjusted for PA, SB as inactivity, etc.) |
| Bock (2019) | http://dx.doi.org/10.1016/j.amepre.2018.11.026 | Population chosen with high risk of type 2 diabetes |
| Bock (2019) | http://dx.doi.org/10.1016/j.diabres.2019.06.011 | Wrong exposures (SB not reported, SB not adjusted for PA, SB as inactivity, etc.) |
| Bodker (2017) | https://www.ahajournals.org/doi/10.1161/circ.136.suppl_1.19398 | Abstract only |
| Bodker (2021) | http://dx.doi.org/10.1177/1358863X211001934 | BMI > 30 |
| Bohn (2017) | http://dx.doi.org/10.1055/s-0043-101676 | Wrong outcomes |
| BowdenDavies (2021) | http://dx.doi.org/10.3389/fphys.2021.659834 | Wrong exposures (SB not reported, SB not adjusted for PA, SB as inactivity, etc.) |
| Boyer (2016) | http://dx.doi.org/10.5888/pcd13.160159 | Wrong exposures (SB not reported, SB not adjusted for PA, SB as inactivity, etc.) |
| Brage (2004) | http://dx.doi.org/10.2337/diacare.27.9.2141 | Paedriatic population (<18) |
| Brage (2004) | http://dx.doi.org/10.1038/sj.ijo.0802772 | Paedriatic population (<18) |
| Brito (2014) | http://dx.doi.org/10.1111/obr.12151 | Paedriatic population (<18) |
| Buman (2012) | http://dx.doi.org/10.1016/j.jsams.2012.11.183 | Abstract only |
| Buman (2016) | https://dx.doi.org/10.1155/2016/4856506 | Population chosen with high risk of type 2 diabetes |
| Butte (2006) | https://doi.org/10.1093/ajcn/84.3.646 | Paedriatic population (<18) |
| Butte (2007) | http://dx.doi.org/10.1249/mss.0b013e3180621fb6 | Paedriatic population (<18) |
| Cadenas-Sanchez (2017) | http://dx.doi.org/10.1016/j.jadohealth.2017.01.018 | Paedriatic population (<18) |
| Carlson (2014) | https://www.ahajournals.org/doi/10.1161/circ.129.suppl_1.p429 | Paedriatic population (<18) |
| Carr (2008) | https://dx.doi.org/10.1016/j.ypmed.2007.12.005 | Wrong activity measurement (subjective or non-accelerometer) |
| Carson (2011) | http://dx.doi.org/10.1186/1471-2458-11-274 | Paedriatic population (<18) |
| Carson (2017) | http://dx.doi.org/10.1016/j.ypmed.2016.12.005 | Paedriatic population (<18) |
| Cassidy (2018) | http://dx.doi.org/10.1007/s00592-018-1161-8 | Wrong exposures (SB not reported, SB not adjusted for PA, SB as inactivity, etc.) |
| Celis-Morales (2013) | http://dx.doi.org/10.1371/journal.pone.0082568 | Wrong exposures (SB not reported, SB not adjusted for PA, SB as inactivity, etc.) |
| Chase (2014) | https://doi.org/10.25011/cim.v37i2.21093 | Geriatric population (>65) |
| Chen (2020) | http://dx.doi.org/10.1093/ajcn/nqaa232 | Wrong exposures (SB not reported, SB not adjusted for PA, SB as inactivity, etc.) |
| Chevalier (2010) | Chevalier, Stephanie, et al. "Healthy Aging Is Associated with Normal Insulin Sensitivity and Protein Anabolic Response to Feeding." DIABETES . Vol. 59. 1701 N BEAUREGARD ST, ALEXANDRIA, VA 22311-1717 USA: AMER DIABETES ASSOC, 2010. | Abstract only |
| Choi (2016) | http://dx.doi.org/10.1017/S1368980015003742 | Wrong activity measurement (subjective or non-accelerometer) |
| Christensen (2009) | http://dx.doi.org/10.1016/j.diabres.2009.03.007 | Wrong exposures (SB not reported, SB not adjusted for PA, SB as inactivity, etc.) |
| Christensen (2012) | http://dx.doi.org/10.1002/ajhb.22239 | Wrong exposures (SB not reported, SB not adjusted for PA, SB as inactivity, etc.) |
| Clamp (2017) | http://dx.doi.org/10.1038/nutd.2017.31 | Wrong exposures (SB not reported, SB not adjusted for PA, SB as inactivity, etc.) |
| Clarke (2014) | https://dx.doi.org/10.1249/MSS.0b013e31829f83a0 | Wrong exposures (SB not reported, SB not adjusted for PA, SB as inactivity, etc.) |
| Colpani (2013) | https://dx.doi.org/10.1097/GME.0b013e318271b388 | Wrong activity measurement (subjective or non-accelerometer) |
| Colpitts (2021) | http://dx.doi.org/10.1002/tsm2.216 | Wrong outcomes |
| Cononie (1994) | https://doi.org/10.1111/j.1532-5415.1994.tb07487.x | Geriatric population (>65) |
| Deenik (2017) | http://dx.doi.org/10.1007/s00406-017-0824-8 | Not in English |
| DeJong (2018) | http://dx.doi.org/10.3390/ijerph15112566 | BMI > 30 |
| deLaMaza (2014) | https://dx.doi.org/10.3305/nh.2015.31.3.7979 | Not in English |
| deLaMaza (2015) | http://dx.doi.org/10.3305/nh.2015.31.3.7979 | Population chosen with type 2 diabetes at baseline |
| DeRooij (2016) | http://dx.doi.org/10.1371/journal.pone.0154358 | Population with underlying health conditions |
| DeWinter (2018) | http://dx.doi.org/10.1155/2018/7496768 | Population chosen with high risk of type 2 diabetes |
| DiBlasio (2018) | http://dx.doi.org/10.23736/S0022-4707.17.07320-0 | Wrong outcomes |
| Dickie (2013) | https://doi.org/10.1080/22201009.2013.10872303 | Wrong activity measurement (subjective or non-accelerometer) |
| Dickie (2016) | http://dx.doi.org/10.1089/met.2015.0064 | Abstract only |
| Diniz (2015) | http://dx.doi.org/10.1016/j.ejogrb.2015.09.013 | Wrong exposures (SB not reported, SB not adjusted for PA, SB as inactivity, etc.) |
| Diniz (2017) | http://dx.doi.org/10.1590/2359-3997000000259 | Geriatric population (>65) |
| DiRaimondo (2013) | http://dx.doi.org/10.1111/ijcp.12269 | Wrong exposures (SB not reported, SB not adjusted for PA, SB as inactivity, etc.) |
| Drigny (2014) | http://dx.doi.org/10.2340/16501977-1905 | Population chosen with high risk of type 2 diabetes |
| Dumuid (2018) | http://dx.doi.org/10.1016/j.maturitas.2018.02.003 | Geriatric population (>65) |
| Duvivier (2013) | http://dx.doi.org/10.1371/journal.pone.0055542 | Wrong study design (not observational) |
| Duvivier (2017) | http://dx.doi.org/10.3389/fphys.2017.00353 | BMI > 30 |
| Duvivier (2018) | https://dx.doi.org/10.1038/s41598-018-26616-w | Wrong study design (not observational) |
| Edwards (2012) | http://dx.doi.org/10.1016/j.metabol.2011.10.016 | Paedriatic population (<18) |
| Edwards (2018) | http://dx.doi.org/10.1177/0890117116684889 | Wrong outcomes |
| Edwardson (2012) | http://dx.doi.org/10.1016/j.jsams.2012.11.397 | Abstract only |
| Edwardson (2017) | https://dx.doi.org/10.1136/bmjopen-2016-014267 | Population chosen with high risk of type 2 diabetes |
| Edwardson (2020) | 10.1249/MSS.0000000000002138 | Population chosen with high risk of type 2 diabetes |
| Ekblom (2015) | https://dx.doi.org/10.1371/journal.pone.0131586 | Wrong outcomes |
| Ekelund (2005) | http://dx.doi.org/10.2337/diacare.28.5.1195 | Wrong activity measurement (subjective or non-accelerometer) |
| Ekelund (2007) | http://dx.doi.org/10.2337/dc06-1883 | Population chosen with high risk of type 2 diabetes |
| Ekelund (2008) | https://doi.org/10.1093/ajcn/88.3.612 | Wrong activity measurement (subjective or non-accelerometer) |
| Ekelund (2009) | http://dx.doi.org/10.2337/dc08-1895 | Population chosen with high risk of type 2 diabetes |
| Ellery (2014) | http://dx.doi.org/10.1038/ijo.2013.125 | Paedriatic population (<18) |
| Eppeland (2013) | http://dx.doi.org/10.1136/annrheumdis-2012-eular.1765 | Population with underlying health conditions |
| Escolar (2009) | http://dx.doi.org/10.1016/j.gaceta.2008.12.005 | Not in English |
| Evans (1995) | https://doi.org/10.1093/gerona/50a.special_issue.147 | Geriatric population (>65) |
| Evans (1997) | https://doi.org/10.1093/jn/127.5.998s | Geriatric population (>65) |
| Evans (1999) | http://dx.doi.org/10.1097/00005768-199901000-00004 | Geriatric population (>65) |
| Ewald (2012) | http://dx.doi.org/10.1016/j.jsams.2012.11.581 | Abstract only |
| Ewald (2014) | http://dx.doi.org/10.1123/jpah.2012-0091 | Geriatric population (>65) |
| Farah (2016) | http://dx.doi.org/10.1016/j.jvs.2015.09.018 | Population with underlying health conditions |
| Farni (2014) | https://dx.doi.org/10.7717/peerj.499 | Wrong exposures (SB not reported, SB not adjusted for PA, SB as inactivity, etc.) |
| Ferriolli (2017) | http://dx.doi.org/10.1159/000480486 | Abstract only |
| Figueiro (2019) | http://dx.doi.org/10.1371/journal.pone.0210861 | Geriatric population (>65) |
| Forde (2018) | http://dx.doi.org/10.1007/s10461-017-1715-8 | Population with underlying health conditions |
| Franks (2002) | http://dx.doi.org/10.1079/BJN2002663 | Wrong activity measurement (subjective or non-accelerometer) |
| Franks (2004) | http://dx.doi.org/10.2337/diacare.27.5.1187 | Wrong activity measurement (subjective or non-accelerometer) |
| Freak-Poli (2011) | http://dx.doi.org/10.1016/j.ypmed.2011.06.005 | Wrong activity measurement (subjective or non-accelerometer) |
| Freak-Poli (2013) | http://dx.doi.org/10.1002/oby.20342 | Wrong activity measurement (subjective or non-accelerometer) |
| Freene (2018) | http://dx.doi.org/10.1097/HCR.0000000000000334 | Population with underlying health conditions |
| Fretts (2012) | http://dx.doi.org/10.2337/dc11-2321 | Wrong activity measurement (subjective or non-accelerometer) |
| Full (2018) | https://www.ahajournals.org/doi/10.1161/circ.137.suppl_1.p349 | Abstract only |
| Full (2019) | http://dx.doi.org/10.1161/circ.139.suppl_1.022 | Geriatric population (>65) |
| Full (2021) | http://dx.doi.org/10.1016/j.ypmed.2021.106626 | Wrong outcomes |
| Gany (2014) | http://dx.doi.org/10.1007/s11524-013-9858-z | Wrong activity measurement (subjective or non-accelerometer) |
| Garcia-Hermoso (2016) | http://dx.doi.org/10.1016/j.ypmed.2015.11.012 | Wrong exposures (SB not reported, SB not adjusted for PA, SB as inactivity, etc.) |
| Gay (2014) | http://dx.doi.org/10.1123/jpah.2012-0174 | Wrong exposures (SB not reported, SB not adjusted for PA, SB as inactivity, etc.) |
| Gay (2016) | http://dx.doi.org/10.1016/j.ypmed.2016.01.008 | Wrong exposures (SB not reported, SB not adjusted for PA, SB as inactivity, etc.) |
| Gennuso (2013) | http://dx.doi.org/10.1249/MSS.0b013e318288a1e5 | Geriatric population (>65) |
| Glazer (2013) | http://dx.doi.org/10.1249/MSS.0b013e31826beae5 | Wrong exposures (SB not reported, SB not adjusted for PA, SB as inactivity, etc.) |
| Godino (2012) | http://dx.doi.org/10.2337/db12-1-377 | Abstract only |
| Godino (2016) | http://dx.doi.org/10.1371/journal.pmed.1002185 | Wrong exposures (SB not reported, SB not adjusted for PA, SB as inactivity, etc.) |
| Green (2014) | https://doi.org/10.1007/s00421-014-2822-0 | Wrong outcomes |
| Hallsworth (2012) | http://dx.doi.org/10.1111/j.1464-5491.2011.03555_1.x | Abstract only |
| Hallsworth (2013) | http://dx.doi.org/10.1016/S0168-8278%2813%2961338-0 | Wrong outcomes |
| Hallsworth (2015) | http://dx.doi.org/10.1136/flgastro-2014-100432 | Population with underlying health conditions |
| Hamer (2013) | https://dx.doi.org/10.1007%2Fs00125-013-3051-5 | Population chosen with type 2 diabetes at baseline |
| Hamer (2014) | http://dx.doi.org/10.1249/MSS.0000000000000317 | Geriatric population (>65) |
| Hargens (2014) | https://dx.doi.org/10.3978/j.issn.2223-3652.2014.12.06 | Wrong outcomes |
| Hautala (2015) | http://dx.doi.org/10.1016/j.physio.2015.03.3360 | Population with underlying health conditions |
| Hawkins (2014) | http://dx.doi.org/10.1177/1358863X14536630 | Wrong outcomes |
| Hazizi (2012) | https://nutriweb.org.my/mjn/2012.php | Wrong outcomes |
| He (2011) | https://doi.org/10.1093/edrv/32.supp.1 | Abstract only |
| Healy (2008) | http://dx.doi.org/10.2337/dc07-2046 | Wrong exposures (SB not reported, SB not adjusted for PA, SB as inactivity, etc.) |
| Healy (2008) | http://dx.doi.org/10.2337/dc07-1795 | Wrong exposures (SB not reported, SB not adjusted for PA, SB as inactivity, etc.) |
| Healy (2014) | http://dx.doi.org/10.1016/j.jsams.2014.11.236 | Abstract only |
| Helmerhorst (2009) | http://dx.doi.org/10.2337/db08-1773 | Wrong activity measurement (subjective or non-accelerometer) |
| Henson (2012) | http://dx.doi.org/10.1016/j.jsams.2012.11.488 | Population chosen with high risk of type 2 diabetes |
| Henson (2013) | http://dx.doi.org/10.1007/s00125-013-2845-9 | Population chosen with high risk of type 2 diabetes |
| Henson (2013) | http://dx.doi.org/10.1371/journal.pone.0078350 | Population chosen with high risk of type 2 diabetes |
| Henson (2018) | https://dx.doi.org/10.1080/02640414.2017.1405709 | Population chosen with high risk of type 2 diabetes |
| Herzig (2014) | http://dx.doi.org/10.1038/ijo.2013.224 | Population chosen with high risk of type 2 diabetes |
| Hjellset (2009) | http://dx.doi.org/10.1111/j.1753-0407.2009.00020.x | Abstract only |
| Hjellset (2011) | https://www.ntnu.no/ojs/index.php/norepid/issue/view/187 | Not in English |
| Honda (2016) | https://dx.doi.org/10.1186/s12889-016-3570-3 | Wrong outcomes |
| Hornberg (2018) | http://dx.doi.org/10.1136/annrheumdis-2018-eular.3930 | Abstract only |
| Horner (2016) | http://dx.doi.org/10.1017/S0029665116001142 | Abstract only |
| Hou (2018) | http://dx.doi.org/10.1002/dmrr.2963 | Wrong exposures (SB not reported, SB not adjusted for PA, SB as inactivity, etc.) |
| Howard (2014) | http://dx.doi.org/10.1016/j.jsams.2014.11.238 | Abstract only |
| Howard (2015) | http://dx.doi.org/10.1249/MSS.0000000000000631 | Wrong exposures (SB not reported, SB not adjusted for PA, SB as inactivity, etc.) |
| Hult (2019) | http://dx.doi.org/10.2337/cd18-0041 | Geriatric population (>65) |
| Hurtig-Wennlof (2010) | http://dx.doi.org/10.1017/S1368980010000157 | Geriatric population (>65) |
| Imayama (2010) | https://doi.org/10.1186/1479-5868-8-118 | BMI > 30 |
| Jardim (2015) | http://dx.doi.org/10.1186/s12889-015-2477-8 | Wrong activity measurement (subjective or non-accelerometer) |
| Jefferis (2016) | http://dx.doi.org/10.1186/s12966-016-0361-2 | Geriatric population (>65) |
| Jimenez (2012) | http://dx.doi.org/10.1016/j.jsams.2012.11.574 | Abstract only |
| Jorgensen (2016) | http://dx.doi.org/10.1007/s00125-016-4046-9 | Abstract only |
| Jurimae (2010) | http://dx.doi.org/10.1007/s00421-010-1423-9 | Geriatric population (>65) |
| Kalmi (2012) | http://dx.doi.org/10.3923/pjn.2012.523.528 | Wrong exposures (SB not reported, SB not adjusted for PA, SB as inactivity, etc.) |
| Kanagasabai (2017) | http://dx.doi.org/10.1089/met.2016.0103 | Wrong exposures (SB not reported, SB not adjusted for PA, SB as inactivity, etc.) |
| Khoja (2016) | http://dx.doi.org/10.1002/acr.22711 | Population with underlying health conditions |
| Kim (2011) | https://dx.doi.org/10.1186/1471-2458-11-624 | Wrong exposures (SB not reported, SB not adjusted for PA, SB as inactivity, etc.) |
| Kim (2013) | http://dx.doi.org/10.1620/tjem.229.11 | Wrong activity measurement (subjective or non-accelerometer) |
| Kingsnorth (2018) | https://dx.doi.org/10.2196/mhealth.9471 | Wrong exposures (SB not reported, SB not adjusted for PA, SB as inactivity, etc.) |
| Knaeps (2018) | http://dx.doi.org/10.1136/bjsports-2016-096083 | Wrong activity measurement (subjective or non-accelerometer) |
| Knight (2013) | http://dx.doi.org/10.1016/j.jcjd.2013.08.142 | Abstract only |
| Knight (2014) | http://dx.doi.org/10.3810/psm.2014.09.2080 | Geriatric population (>65) |
| Koniak-Griffin (2014) | http://dx.doi.org/10.1007/s10903-013-9782-z | Wrong exposures (SB not reported, SB not adjusted for PA, SB as inactivity, etc.) |
| Kozakova (2011) | http://dx.doi.org/10.1093/eurheartj/ehr325 | Abstract only |
| Kozakova (2013) | http://dx.doi.org/10.1007/s00380-011-0215-4 | Wrong exposures (SB not reported, SB not adjusted for PA, SB as inactivity, etc.) |
| Kulinski (2018) | http://dx.doi.org/10.1136/jim-2018-000745.1 | Abstract only |
| LaCroix (2017) | http://dx.doi.org/10.1186/s12889-017-4065-6 | Geriatric population (>65) |
| Lakoski (2014) | http://dx.doi.org/10.1249/MSS.0000000000000211 | Wrong exposures (SB not reported, SB not adjusted for PA, SB as inactivity, etc.) |
| LeCheminant (2011) | http://dx.doi.org/10.1177/0145721711411108 | Wrong exposures (SB not reported, SB not adjusted for PA, SB as inactivity, etc.) |
| Leiva (2017) | https://dx.doi.org/10.4067/S0034-98872017000400006 | Not in English |
| Lin (2018) | http://dx.doi.org/10.1097/JOM.0000000000001299 | Wrong study design (not observational) |
| Liu (2015) | https://dx.doi.org/10.1002/acr.22587 | Population with underlying health conditions |
| Lopez-Martinez (2013) | https://doi.org/10.1123/ijsnem.23.4.312 | Wrong exposures (SB not reported, SB not adjusted for PA, SB as inactivity, etc.) |
| Loprinzi (2013) | http://dx.doi.org/10.4278/ajhp.110916-QUAN-348 | Wrong exposures (SB not reported, SB not adjusted for PA, SB as inactivity, etc.) |
| Loprinzi (2013) | http://dx.doi.org/10.1159/000354752 | Wrong exposures (SB not reported, SB not adjusted for PA, SB as inactivity, etc.) |
| Loprinzi (2014) | https://dx.doi.org/10.1016/j.dhjo.2014.05.005 | Population with underlying health conditions |
| Loprinzi (2015) | http://dx.doi.org/10.1016/j.physbeh.2015.01.002 | Wrong exposures (SB not reported, SB not adjusted for PA, SB as inactivity, etc.) |
| Luke (2011)BMJ | http://dx.doi.org/10.1038/oby.2011.225 | Wrong exposures (SB not reported, SB not adjusted for PA, SB as inactivity, etc.) |
| Luke (2011)Obesity | http://dx.doi.org/10.1186/1471-2458-11-387 | Abstract only |
| Lyden (2015) | http://dx.doi.org/10.1249/MSS.0000000000000499 | Wrong exposures (SB not reported, SB not adjusted for PA, SB as inactivity, etc.) |
| Lynch (2011) | http://dx.doi.org/10.1093/aje/kwr181 | Abstract only |
| MacAnaney (2013) | https://dx.doi.org/10.1016/j.artres.2013.10.097 | Abstract only |
| Maddison (2016) | http://dx.doi.org/10.1016/j.jsams.2015.08.001 | Wrong exposures (SB not reported, SB not adjusted for PA, SB as inactivity, etc.) |
| Madiha (2013) | https://doi.org/10.4158/endp.19.1.j0hx5p4622v5135h | Paedriatic population (<18) |
| Magutah (2018) | https://dx.doi.org/10.1136/bmjsem-2017-000316 | Geriatric population (>65) |
| Mankowski (2015) | https://dx.doi.org/10.1016/j.exger.2015.06.018 | Geriatric population (>65) |
| Manns (2015) | http://dx.doi.org/10.1016/j.@physio.2015.03.1801 | Abstract only |
| Manohar (2011) | http://dx.doi.org/10.2337/db11-1-378 | Abstract only |
| Manohar (2013) | http://dx.doi.org/10.1089/dia.2013.0044 | Abstract only |
| Manohar (2013) | Physical Activity Sensors Are Superior To Heart Rate Monitoring For Real Time Activity Detection: Implications For Closed Loop Diabetes Control | Wrong study design (not observational) |
| Marques-Vidal (2011) | http://dx.doi.org/10.1515/CCLM.2011.738 | Wrong exposures (SB not reported, SB not adjusted for PA, SB as inactivity, etc.) |
| Martinez-Vizcano (2010) | http://dx.doi.org/10.1111/j.1467-789X.2010.00763-7.x | Abstract only |
| Matricciani (2021) | http://dx.doi.org/10.1016/j.sleep.2020.12.001 | Wrong outcomes |
| McCarthy (2017) | http://dx.doi.org/10.1249/MSS.0000000000001218 | Population chosen with high risk of type 2 diabetes |
| McGarrigle (2017) | http://dx.doi.org/10.1158/1538-7445.SABCS16-P3-09-02 | Abstract only |
| McGregor (2019) | http://dx.doi.org/10.1016/j.pmedr.2018.11.006 | Wrong outcomes |
| Midhet (2010) | https://smj.org.sa/content/31/7/768 | Wrong activity measurement (subjective or non-accelerometer) |
| Mielke (2021) | https://doi.org/10.1249/mss.0000000000002696 | Wrong exposures (SB not reported, SB not adjusted for PA, SB as inactivity, etc.) |
| Mikio (2010) | 10.1097/01.hjh.0000379352.27237.83 | Abstract only |
| Mitchell (2018) | http://dx.doi.org/10.1016/j.jsams.2018.05.002 | Population chosen with high risk of type 2 diabetes |
| Morrell (2013) | http://dx.doi.org/10.1089/met.2013.0011 | Wrong activity measurement (subjective or non-accelerometer) |
| Mundle (2016) | http://dx.doi.org/10.1097/HCR.0000000000000217 | Population with underlying health conditions |
| Musto (2010) | http://dx.doi.org/10.1123/jpah.7.6.737 | Wrong activity measurement (subjective or non-accelerometer) |
| Nightingale (2017) | http://dx.doi.org/10.1016/j.apmr.2017.08.059 | Population with underlying health conditions |
| Nilsson (2017) | http://dx.doi.org/10.1371/journal.pone.0175496 | Geriatric population (>65) |
| Nilsson (2018) | http://dx.doi.org/10.1249/MSS.0000000000001582 | Geriatric population (>65) |
| Nur (2018) | http://dx.doi.org/10.3329/bjms.v17i1.35280 | Wrong activity measurement (subjective or non-accelerometer) |
| Nygaard (2017) | http://dx.doi.org/10.1139/apnm-2016-0467 | Population chosen with high risk of type 2 diabetes |
| Park (2008) | http://dx.doi.org/10.1093/gerona/63.10.1119 | Geriatric population (>65) |
| Parker (2017) | http://dx.doi.org/10.1111/%28ISSN%291445-5994 | Abstract only |
| Parsons (2017) | 10.1249/MSS.0000000000001113 | Wrong exposures (SB not reported, SB not adjusted for PA, SB as inactivity, etc.) |
| Peden (2017) | http://dx.doi.org/10.1016/S0735-1097%2817%2935164-1 | Abstract only |
| Pesola (2017) | http://dx.doi.org/10.1371/journal.pone.0183299 | Wrong exposures (SB not reported, SB not adjusted for PA, SB as inactivity, etc.) |
| Peterman (2019) | http://dx.doi.org/10.1123/jpah.2018-0062 | Wrong study design (not observational) |
| Peterson (2014) | http://dx.doi.org/10.1016/j.ypmed.2013.12.014 | Population with underlying health conditions |
| Petrov (2015) | http://dx.doi.org/10.1007/s00125-015-3687-4 | Abstract only |
| Phillips (2018) | http://dx.doi.org/10.1249/MSS.0000000000001511 | Wrong outcomes |
| Pillay (2015) | http://dx.doi.org/10.1186/s12889-015-1381-6 | Wrong activity measurement (subjective or non-accelerometer) |
| Pollock (2018) | http://dx.doi.org/10.1111/sms.13071 | Geriatric population (>65) |
| Pomeroy (2011) | 10.1161/circ.124.suppl\_21.A13378 | Abstract only |
| Pope (2020) | 10.1249/MSS.0000000000002259 | Wrong outcomes |
| Prioreschi (2013)AnnalsoftheRheumaticDiseases | http://dx.doi.org/10.1136/annrheumdis-2013-eular.1026 | Population with underlying health conditions |
| Prioreschi (2013)ClinicalRheumatology | http://dx.doi.org/10.1007/s10067-013-2314-3 | Population with underlying health conditions |
| Qader (2008) | http://dx.doi.org/10.1080/13697130802451787 | Wrong exposures (SB not reported, SB not adjusted for PA, SB as inactivity, etc.) |
| Qi (2015) | 10.1161/circ.131.suppl_1.36 | Geriatric population (>65) |
| Ramirez-Marrero (2014) | http://prhsj.rcm.upr.edu/index.php/prhsj/article/view/992 | Population with underlying health conditions |
| Randers (2012) | http://dx.doi.org/10.1007/s00421-011-2171-1 | Wrong exposures (SB not reported, SB not adjusted for PA, SB as inactivity, etc.) |
| Rao (2016) | https://dx.doi.org/10.1186/s12966-016-0350-5 | Wrong outcomes |
| Rava (2019) | http://dx.doi.org/10.1007/s40520-019-01222-6 | Geriatric population (>65) |
| Roberts (2013) | http://dx.doi.org/10.1016/j.metabol.2012.12.004 | BMI > 30 |
| Robson (2015) | http://dx.doi.org/10.7717/peerj.1437 | Wrong exposures (SB not reported, SB not adjusted for PA, SB as inactivity, etc.) |
| Rockette-Wagner (2014) | http://dx.doi.org/10.2337/db14-1317-1629 | Abstract only |
| Ross (2015) | http://dx.doi.org/10.7326/M14-1189 | Population chosen with high risk of type 2 diabetes |
| Rotberg (2012) | http://dx.doi.org/10.2337/db12-656-835 | Population chosen with type 2 diabetes at baseline |
| Rutters (2015) | http://dx.doi.org/10.1007/s00125-015-3687-4 | Abstract only |
| Rutters (2016) | http://dx.doi.org/10.1210/jc.2016-1045 | Wrong exposures (SB not reported, SB not adjusted for PA, SB as inactivity, etc.) |
| Ryan (2014) | https://dx.doi.org/10.1016/j.ridd.2014.03.051 | Population with underlying health conditions |
| Sachdev (2015) | http://dx.doi.org/10.1159/000437032 | Paedriatic population (<18) |
| Saibandith (2016) | http://dx.doi.org/10.1017/S0029665116002251 | Wrong activity measurement (subjective or non-accelerometer) |
| Salas (2016) | https://dx.doi.org/10.4067/S0034-98872016001100005 | Wrong exposures (SB not reported, SB not adjusted for PA, SB as inactivity, etc.) |
| Salas-Salvado (2014) | https://doi.org/10.7326/m13-1725 | Abstract only |
| Salonen (2015) | http://dx.doi.org/10.1371/journal.pone.0126737 | Wrong exposures (SB not reported, SB not adjusted for PA, SB as inactivity, etc.) |
| Sandbakk (2016) | http://dx.doi.org/10.1016/j.mayocp.2016.07.020 | Geriatric population (>65) |
| Saunders (2015) | https://doi.org/10.1016/j.jcjd.2015.01.248 | Population with underlying health conditions |
| Schmidt (2009) | https://dx.doi.org/10.1016/j.amepre.2009.05.020 | Wrong activity measurement (subjective or non-accelerometer) |
| Schwenke (2014) | http://dx.doi.org/10.2337/db14-1317-1629 | Abstract only |
| Sepah (2014) | https://doi.org/10.1177/0145721714531339 | Wrong study design (not observational) |
| Serrano (2013) | http://dx.doi.org/10.1096/fasebj.27.1_supplement.845.9 | Abstract only |
| Shin (2017) | http://dx.doi.org/10.1097/JCN.0000000000000406 | Population chosen with high risk of type 2 diabetes |
| Siddiqui (2017) | http://dx.doi.org/10.1007/s00125-017-4350-z | Abstract only |
| Siddiqui (2018) | http://dx.doi.org/10.1016/j.amepre.2018.04.016 | Population chosen with high risk of type 2 diabetes |
| Siddiqui (2018) | http://dx.doi.org/10.1080/16089677.2018.1515144 | Population chosen with type 2 diabetes at baseline |
| Simmons (2008) | http://dx.doi.org/10.1007/s00125-008-0949-4 | Wrong exposures (SB not reported, SB not adjusted for PA, SB as inactivity, etc.) |
| Sjoros (2020) | http://dx.doi.org/10.1038/s41598-020-77637-3 | BMI > 30 |
| Sjoros (2021) | http://dx.doi.org/10.3390/ijerph18094950 | BMI > 30 |
| Slobodova (2017) | https://casopisvnitrnilekarstvi.cz/pdfs/vnl/2017/89/16.pdf | Geriatric population (>65) |
| Spartano (2015) | https://doi.org/10.1161/circ.132.suppl_3.19204 | Abstract only |
| Stuckey (2011) | https://dx.doi.org/10.1177%2F193229681100500416 | Wrong activity measurement (subjective or non-accelerometer) |
| Suboc (2016) | http://dx.doi.org/10.1093/ajh/hpv063 | Geriatric population (>65) |
| Swindell (2018) | http://dx.doi.org/10.2337/dc17-1057 | Population chosen with high risk of type 2 diabetes |
| Thomas (2018) | http://dx.doi.org/10.1097/PSY.0000000000000578 | Abstract only |
| Thomsen (2016)AnnalsoftheRheumaticDiseases | http://dx.doi.org/10.1002/art.39977 | Abstract only |
| Thomsen (2016)ArthritisandRheumatology | http://dx.doi.org/10.1136/annrheumdis-2016-eular.4668 | Population with underlying health conditions |
| Thomsen (2018) | http://dx.doi.org/10.1136/annrheumdis-2018-eular.4595 | Population with underlying health conditions |
| Tierney (2013) | http://dx.doi.org/10.1136/annrheumdis-2013-eular.1719 | Population with underlying health conditions |
| Tigbe (2009) | http://dx.doi.org/10.1111/j.1753-0407.2009.00019.x | Abstract only |
| Tigbe (2011) | http://dx.doi.org/10.1111/j.1467-789X.2011.00877.x | Abstract only |
| Tigbe (2017) | http://dx.doi.org/10.1038/ijo.2017.30 | Wrong outcomes |
| Tucker (2016) | http://dx.doi.org/10.4278/ajhp.121127-QUAN-576 | Wrong exposures (SB not reported, SB not adjusted for PA, SB as inactivity, etc.) |
| Tudor-Locke (2017) | http://dx.doi.org/10.1249/MSS.0000000000001100 | Wrong exposures (SB not reported, SB not adjusted for PA, SB as inactivity, etc.) |
| Ukropec (2017) | https://aanddjournal.net/article/S1552-5260(17)30902-0/pdf | Abstract only |
| VanDerBerg (2015)Diabetologia | http://dx.doi.org/10.1007/s00125-015-3687-4 | Abstract only |
| VanDerBerg (2015)EJE | http://dx.doi.org/10.1007/s10654-015-0072-z | Abstract only |
| VanDerBerg (2017) | 10.1249/MSS.0000000000001248 | Wrong exposures (SB not reported, SB not adjusted for PA, SB as inactivity, etc.) |
| VanDerPloeg (2010) | https://doi.org/10.1080/02701367.2010.10599632 | Wrong study design (not observational) |
| VarelaMato (2016) | http://dx.doi.org/10.1177/2047487316668128 | Abstract only |
| Vasquez (2021) | http://dx.doi.org/10.1177/1540415320985581 | Wrong exposures (SB not reported, SB not adjusted for PA, SB as inactivity, etc.) |
| Veerabhadrappa (2013) | https://doi.org/10.1161/hyp.62.suppl_1.A260 | Abstract only |
| Vella (2011) | https://doi.org/10.1080/02640414.2010.520727 | Wrong exposures (SB not reported, SB not adjusted for PA, SB as inactivity, etc.) |
| Vella (2020) | http://dx.doi.org/10.1007/s10865-020-00161-2 | Wrong activity measurement (subjective or non-accelerometer) |
| Viskochil (2012) | http://dx.doi.org/10.2337/db12-656-835 | Abstract only |
| Weinhold (2015) | http://dx.doi.org/10.5888/pcd12.150301 | Population chosen with high risk of type 2 diabetes |
| Welin (2003) | http://dx.doi.org/10.1097/00149831-200308000-00011 | Geriatric population (>65) |
| Wientzek (2014) | http://dx.doi.org/10.1123/ijsnem.2013-0048 | Wrong outcomes |
| Wijndaele (2010) | 10.1249/MSS.0b013e3181d322ac | Wrong activity measurement (subjective or non-accelerometer) |
| Wijndaele (2012) | http://dx.doi.org/10.1016/j.jsams.2012.11.491 | Abstract only |
| Wijndaele (2014) | http://dx.doi.org/10.1007/s00125-013-3102-y | Population chosen with high risk of type 2 diabetes |
| Wijsman (2013) | https://doi.org/10.2196/jmir.2843 | Geriatric population (>65) |
| Wilson (2015) | http://dx.doi.org/10.1002/ajhb.22625 | Wrong exposures (SB not reported, SB not adjusted for PA, SB as inactivity, etc.) |
| Wirth (2016) | http://dx.doi.org/10.1158/1538-7755.DISP15-A69 | Abstract only |
| Wittekind (2017) | https://www.ahajournals.org/doi/10.1161/circ.135.suppl_1.p308 | Paedriatic population (<18) |
| Wolff-Hughes (2015) | http://dx.doi.org/10.1123/jpah.2013-0463 | Wrong exposures (SB not reported, SB not adjusted for PA, SB as inactivity, etc.) |
| Wolszakiewicz (2015) | http://dx.doi.org/10.5603/KP.a2014.0165 | Population with underlying health conditions |
| Woolf (2008) | http://dx.doi.org/10.1016/j.jada.2008.03.015 | Wrong activity measurement (subjective or non-accelerometer) |
| Woolf (2015) | https://doi.org/10.1096/fasebj.29.1_supplement.588.5 | Population with underlying health conditions |
| Yates (2010) | http://dx.doi.org/10.1111/j.1464-5491.2010.03091.x | Wrong activity measurement (subjective or non-accelerometer) |
| Yates (2012) | http://dx.doi.org/10.1016/j.jsams.2012.11.489 | Abstract only |
| Yates (2015) | http://dx.doi.org/10.1371/journal.pone.0124062 | Population chosen with high risk of type 2 diabetes |
| Yates (2015) | http://dx.doi.org/10.1016/j.ypmed.2015.04.005 | Population chosen with high risk of type 2 diabetes |
| Yates (2020) | 10.1249/MSS.0000000000002204 | Population chosen with high risk of type 2 diabetes |
| Yiallourou (2018) | http://dx.doi.org/10.1111/jsr.12751 | Abstract only |
| Younger (2016) | http://dx.doi.org/10.1080/02640414.2015.1068435 | Wrong study design (not observational) |
| Zecchin (2013) | http://dx.doi.org/10.1089/dia.2012.1221 | Abstract only |
| Zecchin (2013) | https://doi.org/10.1089/dia.2013.0105 | Abstract only |
| Zhou (2016) | http://dx.doi.org/10.1097/JOM.0000000000000673 | Wrong exposures (SB not reported, SB not adjusted for PA, SB as inactivity, etc.) |

# S3 Table. Quality assessment of the selected studies

| **Study** | **Representativeness of the cohort: 1 point if the population does not include individuals with metabolic conditions (diabetes, met syndrome, etc). OK to have diabetes if it is an outcome.** | **Description of the cohort: 1 point if sex, age, BMI/WC and metabolic health reported with mean/median and std dev/quartiles. 1 point only if all 4 reported** | **Ascertainment of exposure: 1 point if the study had at least 4 valid days (>10h) of accelerometer** | **Analysis adjusted for body mass index (BMI) or waist circumference (WC)** | **Analysis adjusted for sex (if males and females combined), age and ethnicity (point given only if adjusted for all 3 or only 1 ethnicity used)** | **Analysis adjusted for accelerometer wear time or expressed exposures in relationship to wear time** | **Presentation of point estimates and measures of variability (confidence interval or standard error)** | **Total** | **Quality** |
| --- | --- | --- | --- | --- | --- | --- | --- | --- | --- |
| Bakrania 2016 | 1 | 1 | 1 | 1 | 1 | 1 | 1 | 7 | High |
| Balkau 2008 | 1 | 1 | 0 | 1 | 0 | 0 | 1 | 4 | Medium |
| BaroneGibbs 2015 | 0 | 0 | 1 | 1 | 1 | 1 | 1 | 5 | Medium |
| Buman 2014 | 0 | 0 | 1 | 0 | 1 | 1 | 1 | 4 | Medium |
| Carson 2014 | 0 | 1 | 1 | 0 | 0 | 0 | 1 | 3 | Poor |
| Celis-Morales 2012 | 0 | 1 | 1 | 0 | 1 | 0 | 1 | 4 | Medium |
| Chastin 2015 | 0 | 1 | 1 | 1 | 1 | 0 | 1 | 5 | Medium |
| Debache 2019 | 0 | 1 | 1 | 1 | 0 | 0 | 1 | 4 | Medium |
| Diaz 2017 | 0 | 1 | 0 | 0 | 1 | 0 | 1 | 3 | Poor |
| Edwards 2018 | 0 | 0 | 1 | 0 | 1 | 0 | 1 | 3 | Poor |
| Ekblom-Bak 2015 | 1 | 1 | 1 | 0 | 0 | 1 | 1 | 5 | Medium |
| Ekblom-Bak 2016 | 0 | 1 | 1 | 0 | 0 | 1 | 1 | 4 | Medium |
| Elhakeem 2018 | 0 | 0 | 0 | 0 | 0 | 1 | 1 | 2 | Poor |
| Farrahi 2021 | 0 | 1 | 1 | 0 | 0 | 0 | 1 | 3 | Poor |
| Farrahi 2021-b | 0 | 1 | 1 | 0 | 0 | 0 | 1 | 3 | Poor |
| Garcia-Hermoso 2015 | 0 | 1 | 1 | 0 | 0 | 1 | 1 | 4 | Medium |
| Gennuso 2014 | 0 | 1 | 0 | 0 | 1 | 1 | 1 | 4 | Medium |
| Gradmark 2011 | 1 | 1 | 1 | 0 | 0 | 1 | 1 | 5 | Medium |
| Healy 2007 | 0 | 1 | 1 | 1 | 0 | 1 | 1 | 5 | Medium |
| Healy 2011 | 0 | 1 | 1 | 1 | 1 | 1 | 1 | 6 | High |
| Healy 2015 | 0 | 1 | 0 | 0 | 1 | 1 | 1 | 4 | Medium |
| Honda 2014 | 1 | 1 | 1 | 0 | 0 | 1 | 1 | 5 | Medium |
| Honda 2019 | 1 | 1 | 1 | 0 | 0 | 1 | 1 | 5 | Medium |
| Kim 2013 | 1 | 0 | 1 | 0 | 1 | 1 | 1 | 5 | Medium |
| Knaeps 2016 | 0 | 0 | 1 | 0 | 0 | 0 | 1 | 2 | Poor |
| Lahjibi 2013 | 1 | 1 | 0 | 0 | 0 | 1 | 1 | 4 | Medium |
| Loprinzi 2014 | 0 | 0 | 1 | 1 | 1 | 1 | 1 | 5 | Medium |
| Lynch 2011 | 1 | 1 | 1 | 1 | 1 | 1 | 1 | 7 | High |
| Maher 2014 | 0 | 0 | 1 | 0 | 1 | 1 | 1 | 4 | Medium |
| McGregor 2018 | 0 | 1 | 1 | 0 | 0 | 0 | 1 | 3 | Poor |
| Mossavar-Rahmani 2020 | 0 | 0 | 0 | 0 | 1 | 1 | 1 | 3 | Poor |
| Parsons 2017 | 0 | 1 | 0 | 1 | 1 | 1 | 1 | 5 | Medium |
| Peterson 2014 | 0 | 1 | 1 | 0 | 0 | 1 | 0 | 3 | Poor |
| Peterson 2015 | 1 | 1 | 1 | 0 | 0 | 1 | 1 | 5 | Medium |
| Phillips 2017 | 0 | 1 | 1 | 1 | 0 | 1 | 1 | 5 | Medium |
| Qi 2015 | 0 | 0 | 0 | 1 | 1 | 1 | 1 | 4 | Medium |
| Scheer 2013 | 0 | 1 | 1 | 0 | 0 | 0 | 1 | 3 | Poor |
| Spartano 2017 | 1 | 1 | 1 | 1 | 1 | 1 | 1 | 7 | High |
| Stamatakis 2012 | 0 | 1 | 0 | 0 | 0 | 1 | 1 | 3 | Poor |
| Stubbs 2017 | 0 | 0 | 0 | 0 | 0 | 0 | 1 | 1 | Poor |
| vanderVelde 2015 | 1 | 1 | 0 | 1 | 1 | 1 | 1 | 6 | High |
| vanderVelde 2018 | 1 | 1 | 0 | 0 | 0 | 0 | 1 | 3 | Poor |
| Varela-Mato 2017 | 0 | 1 | 1 | 1 | 0 | 1 | 1 | 5 | Medium |
| Whitaker 2019 | 0 | 1 | 1 | 1 | 1 | 1 | 1 | 6 | High |
| Zheng 2020 | 1 | 1 | 1 | 1 | 1 | 1 | 1 | 7 | High |
